# A Multitubular Kidney-on-Chip to Decipher Pathophysiological Mechanisms in Renal Cystic Diseases

**DOI:** 10.3389/fbioe.2021.624553

**Published:** 2021-05-26

**Authors:** Sarah Myram, Bastien Venzac, Brice Lapin, Aude Battistella, Fanny Cayrac, Bertrand Cinquin, Charles Cavaniol, Giacomo Gropplero, Isabelle Bonnet, Sophie Demolombe, Stéphanie Descroix, Sylvie Coscoy

**Affiliations:** ^1^Institut Curie, Université PSL (Paris Sciences & Lettres), Sorbonne Université, CNRS UMR 168, Laboratoire Physico Chimie Curie, Paris, France; ^2^Institut Pierre-Gilles de Gennes, IPGG Technology Platform, UMS 3750 CNRS, Paris, France; ^3^Fluigent SA, France; ^4^Université Côte d’Azur, Centre National de la Recherche Scientifique, Institut National de la Santé et de la Recherche Médicale, Institut de Pharmacologie Moléculaire et Cellulaire, Labex ICST, Valbonne, France

**Keywords:** ADPKD, microfabrication, tube deformation, hydrogel, kidney-on-chip

## Abstract

Autosomal Dominant Polycystic Kidney Disease (ADPKD) is a major renal pathology provoked by the deletion of *PKD1* or *PKD2* genes leading to local renal tubule dilation followed by the formation of numerous cysts, ending up with renal failure in adulthood. *In vivo*, renal tubules are tightly packed, so that dilating tubules and expanding cysts may have mechanical influence on adjacent tubules. To decipher the role of this coupling between adjacent tubules, we developed a kidney-on-chip reproducing parallel networks of tightly packed tubes. This original microdevice is composed of cylindrical hollow tubes of physiological dimensions, parallel and closely packed with 100–200 μm spacing, embedded in a collagen I matrix. These multitubular systems were properly colonized by different types of renal cells with long-term survival, up to 2 months. While no significant tube dilation over time was observed with Madin-Darby Canine Kidney (MDCK) cells, wild-type mouse proximal tubule (PCT) cells, or with PCT *Pkd1*^+/-^ cells (with only one functional *Pkd1* allele), we observed a typical 1.5-fold increase in tube diameter with isogenic PCT *Pkd1*^-/-^ cells, an ADPKD cellular model. This tube dilation was associated with an increased cell proliferation, as well as a decrease in F-actin stress fibers density along the tube axis. With this kidney-on-chip model, we also observed that for larger tube spacing, PCT *Pkd1*^-/-^ tube deformations were not spatially correlated with adjacent tubes whereas for shorter spacing, tube deformations were increased between adjacent tubes. Our device reveals the interplay between tightly packed renal tubes, constituting a pioneering tool well-adapted to further study kidney pathophysiology.

## Introduction

Autosomal Dominant Polycystic Kidney Disease (ADPKD) is the most common genetic renal disease (incidence 1/1,000), and the fourth most common cause of end-stage renal failure worldwide, without curative therapies except dialysis or transplantation (Ghata and Cowley, 2017; Li, 2017). It is due to mutations in *PKD1* (85% of cases) or *PKD2* (15% of cases) genes that code for transmembrane proteins, polycystins 1 and 2 (PC1 and PC2), whose expression level is fundamental to maintain the renal epithelium architecture (Lu et al., 1997; Rossetti et al., 2009). Polycystins are involved in many signaling pathways coupled to proliferation, apoptosis, cell cycle, planar polarity and the regulation of cell adhesion and cytoskeleton organization (Chapin and Caplan, 2010; Castelli et al., 2013; Mochizuki et al., 2013; Cornec-Le Gall et al., 2019; Douguet et al., 2019).

ADPKD is characterized by enlarged kidneys in which progressive numerous and bilateral fluid-filled cysts extend from renal tubular epithelial cells (Grantham et al., 1987; Fick et al., 1993; Chapin and Caplan, 2010; Cornec-Le Gall et al., 2019). Fundamental mechanisms involved in cystogenesis are based on increased proliferation of epithelial tubular cells (Terzi et al., 1996; Yamaguchi et al., 2003; Cowley et al., 2006; Grimm et al., 2006; Lee, 2016) coupled with de-regulated apoptosis (Boca et al., 2006; Foy et al., 2012; Kurbegovic and Trudel, 2020), loss of planar polarity and misorientation during mitosis (Fischer et al., 2006; Castelli et al., 2013), and remodeling of extracellular matrix (ECM) (Wilson et al., 1992; Schafer et al., 1994; Ramasubbu et al., 1998; Joly et al., 2003; Subramanian et al., 2012). Relying on those mechanisms, human cysts reaching up to 3 mm in diameter detach from the parent tubule and migrate away while continuing expanding (Ghata and Cowley, 2017). Expanding cysts constrain the functional renal parenchyma, and participate to its progressive failure during ADPKD evolution.

Many studies focused on genetic and molecular factors involved in cystogenesis process during ADPKD (Chapin and Caplan, 2010; Cornec-Le Gall et al., 2014). However, the prominent influence of geometrical and mechanical factors has not yet been investigated. Recent studies on ADPKD patients and specific mice models suggest that primary cysts are randomly formed along the renal tubules, but that secondary cyst formation tends to be spatially clustered. An exponential increase in the probability of secondary cyst formation over time was even reported (Leonhard et al., 2015), in relation with the exponential growth in total kidney volume observed for patients (Grantham et al., 2006, 2008). These cascading events led to the snowball effect theory, relating that primary cyst growth would trigger secondary cyst formation in adjacent tubes and favor abnormal signaling pathways activation within renal epithelial cells. Indeed, neighboring non-cystic tubules were reported to have increased proliferation and apoptosis in the presence of cysts (Nadasdy et al., 1995; Woo, 1995; Grantham et al., 2011).

This snowball effect may be due to chemical coupling between adjacent tubes (El-Achkar and Dagher, 2015), combined or not to local mechanical alterations. Expanding cysts or dilating tubules may exert a mechanical influence on adjacent tubules, either by direct cell compression, or by flow disturbance due to the locally altered shape of tubules (Grantham et al., 2011). It is noteworthy that polycystins are key molecular actors in the control of mechanotransduction in renal tubules (Qian et al., 2005; Patel and Honoré, 2010). Polycystins are present in primary cilia, where their role as direct flow sensors through PC2 channel activity is still debated (Nauli et al., 2003; Delling et al., 2016), as well as in cell-cell and cell-matrix contacts (Huan and van Adelsberg, 1999; Wilson, 2001; Markoff et al., 2007; Lee et al., 2014). They have been centrally involved in mechanosensitive control of cytoskeletal organization and actomyosin contractility (Sharif-Naeini et al., 2009; Bhoonderowa et al., 2016; Nigro et al., 2019). The RhoA-YAP-c-Myc axis has been identified as a key mediator in ADPKD cystogenesis (Happe et al., 2011b; Cai et al., 2018), with YAP mechanosensing (Hippo pathway) playing a central role in the control of the size and shape of tissues and organs.

The geometrical organization of the kidney with densely packed tubules may have a key influence for mechanical or biochemical cross-talks between them. Hence, to decipher the geometrical factors involved in the propagation of deformations between adjacent tubes, we propose here to reproduce those physical properties by developing an array of renal tubules in advanced *in vitro* models called kidney-on-chips. Several microphysiological kidney-on-chips have already been developed to mimic different features of renal tubules, most of them reproducing the proximal tubule-like phenotype and metabolism. The first devices designed were composed of one or two channels in close contact (120–550 μm width) with renal epithelial cells, and were dedicated to the study of renal reabsorption (Jang et al., 2013; Vedula et al., 2017; Lin et al., 2019). More recent works intended to recreate the cylindrical geometry of tubules. Indeed, renal tubules are circular tubes of small diameters (50 μm in the proximal part), and in this range important confinement and curvature effects have been reported for the control of the collective organization of renal cells (Vedula et al., 2012; Yevick et al., 2015). 3D printing techniques were also used to generate circular tubes reproducing the proximal tubule (Homan et al., 2016; Lin et al., 2019), however typically with diameters larger than physiological ones. A versatile technology to generate cylindrical tubes is based on the principle of wire molding (Dolega et al., 2014). It was used to recapitulate the fundamental biochemistry of renal tubular epithelium displaying intracellular enzymatic functions with the vitamin D metabolism (Weber et al., 2016). It was also used to study renal collective dynamics in function of tube diameters (Xi et al., 2017), or to reproduce a change in diameter characteristic for transitions between the different parts of renal tubes (Venzac et al., 2018). While these different approaches have been focused on the study of renal transport function, morphology or collective cell organization, exploring cystic diseases with kidney-on-chips has been scarcely addressed. Recently, microlithography-based approaches were used to generate parallelepiped structures in a collagen-Matrigel matrix, with tube to cyst transition upon cAMP stimulation (Subramanian et al., 2018).

Nevertheless, a kidney-on-chip reproducing the geometry of tightly and cylindrical packed tubules to explore a renal disorder is still missing. In this paper, we report on a microfabrication approach to recapitulate renal tubes of physiological geometries, positioned in parallel with 100 or 200 μm spacing, in a biocompatible and deformable hydrogel. Using this unique device, we studied tube behavior upon seeding with several epithelial renal cell lines, and focused on tube deformation with an ADPKD cellular model.

## Materials and Methods

### Cell Culture

MDCK cells (CCL-34 ATCC, NBL-2), stably expressing Lifeact-GFP, were maintained in DMEM supplemented with 10% FCS and 0.4 mg/ml geneticin at 37°C and 5% CO_2_. Mouse PCT-wild type cells, kindly given by Amanda Patel and Eric Honoré (Peyronnet et al., 2012), were maintained in DMEM/HamF12 (Thermo Fisher Scientific) supplemented with 1% SVF, 15 mM NaHCO_3_, 20 mM HEPES adjusted at pH 7.4 (Thermo Fisher Scientific), 2 mM glutamine, 5 μg/ml insulin (Sigma), 50 nM dexamethasone (Sigma), 1 μg/l EGF (Sigma), 5 mg/l transferrin (Sigma), 30 nM Na selenite (Sigma), 10 nM triiodo-L-thyronine (Sigma) and 125 μg/ml G418 (Sigma), at 37°C, 5% CO_2_. Maintained in a T75 flask, both cell types were split twice a week, when they reached around 70–80% of confluence. All cells were rinsed twice with DPBS (Sigma) and trypsinized with 2 ml of 0.05% Trypsin- ethylenediamine tetraacetic acid (EDTA) (Sigma) at 37°C. All the cell lines were used at a low passage in the different experiments: mostly between passage 6 and 20.

Mouse PCT *Pkd1*^+/-^ and *Pkd1*^-/-^ cells (respectively, PH2 and PN24 clones) were a kind gift of S. Somlo (Joly et al., 2006; Shibazaki et al., 2008; Wei et al., 2008). These cells, containing the Immortomouse transgene for the interferon-inducible expression of a thermolabile large tumor antigen, were amplified in proliferation conditions (33°C, with γ-interferon) and differentiated in differentiation conditions (37°C, without γ-interferon). Proliferation conditions were 33°C, 5% CO_2_, in DMEM/HamF12 supplemented with 3% SVF, 7.5 nM Na selenite, 1.9 nM triiodo-L-thyronine, 5 mg insulin, 5 mg transferrin, 100 UI/ml penicillin/streptomycin, 5 mg/ml nystatin (all from Sigma), and 10 UI/ml γ-interferon (Millipore). Cells were differentiated in the same media without γ-interferon, and with 1% SVF instead of 3% SVF, at 37°C, 5% CO_2_. For 2D immunofluorescence and qRT-PCR experiments, cells were cultured in this medium 7 days before using them in the experiments, at 37°C with 5% CO_2_, to favor cell epithelialization. According to S. Somlo’s group’s specifications, we confirmed by PCR on genomic DNA the presence of a null *Pkd1* allele on exon 1, the insertion of *lox* sites flanking exons 2–4 in one allele of *Pkd1*^+/-^ cells, and the deletion of this floxed *Pkd1* part in *Pkd1*^-/-^ cells.

### qRT-PCR Experiments

2D data correspond to PCT cells differentiated for more than 1 week in differentiation media. Primer sequences, designed with: https://www.ncbi.nlm.nih.gov/tools/primer-blast, were as follow. Primer efficiency was measured on 4 serial dilutions from 1× to 1,000× cDNA. The efficiency E was calculated according to *E* = 10^1/slope^. Efficiencies calculated were between 93 and 111%. mATP1A1 primers were found on Origene (atp1a1-mouse-qpcr-primer-pair-nm_144900). See SI for the sequences of primers.

Total RNA was extracted from differentiated cells using NucleoSpin RNA (Macherey-Nagel). Reverse transcription reactions were performed on 2 μg of total RNA with the high-capacity cDNA reverse transcription kit (Thermo Fisher Scientific) with random hexamers, and mixed with the Applied Biosystems^TM^ PowerUp^TM^ SYBR^TM^ Green Master Mix (Thermo Fisher Scientific) and 5 pmol of both forward and reverse primers (see below). cDNA was diluted 50×. Real-time PCR was carried out using a SteponePlus PCR system (Thermo Fisher Scientific) with the following cycles: 95°C for 10 min (95°C for 15 sec, 60°C for 1 min) × 40 times and read plate. Melting curves from 55 to 95°C (read every 1°C and hold 1 s) were generated. Reactions were run in technical triplicates. Expression data were normalized to the GAPDH housekeeping gene. Analyze used DeltaCt between target and normalizator, then 2^–*DeltaCt*^. Statistical *t*-tests were performed on DeltaCt values. *Pkd1*^-/-^ vs. *Pkd1*^+/-^ fold values are represented.

### Microfabrication and Device Design

**FIGURE 1:**
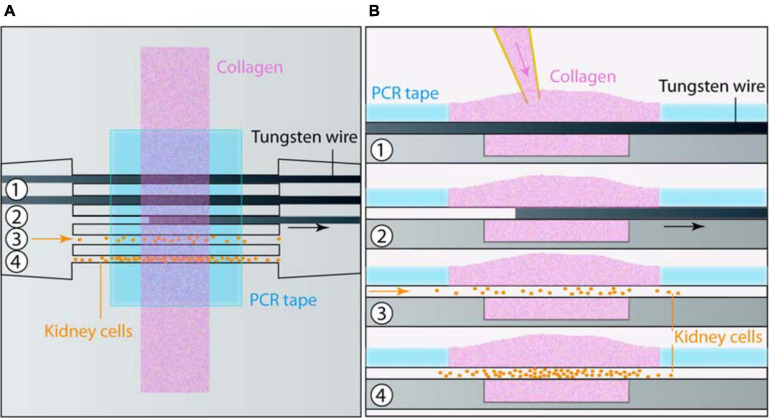
Chip microfabrication and cell seeding. **(A)** Top and **(B)** side views of the sequential steps for microfabrication and cell seeding: (1) Collagen was poured on top of the tungsten wires placed in a micromold and maintained by PCR tape. (2) Wires were removed. At this stage an additional coating could be applied. (3) Cells were seeded in tubes, and (4) colonized it in a few days.

The design of the chip was first drawn with Catya (Dassault Systems, France) and milled on a 50 × 50 × 3 mm brass bloc with a micromilling machine (Minitech, Georgia, United States). The patterns were then transferred via hot embossing (130°C, 7 bars, 10 min) on a 2-mm thick Cyclic Olefin Copolymer (COC, TOPAS 8007-04) plate, a thermoplastic displaying an optimal optical index for the tubes visualization under a microscope (Mottet et al., 2014). On the COC plate, the patterns comprised a rectangular reservoir (1 mm wide, 5 mm long and 300 μm deep) in which the tubes were molded in collagen I, five 1 mm long, 90 μm wide and 90 μm deep grooves on each side of the reservoir, separated by 100 or 200 μm and in which the wires to mold the tubes were positioned (see Figure 1). Two connectors with a semi cylindrical bottom at the side of the COC plate were placed at both extremities of those grooves, with a continuous transition. Liquids and cells were injected through those two connectors.

In order to obtain an adequate covalent adhesion of collagen I, the COC reservoir surface was first treated with an oxygen plasma (Cute, Femto Science, South Korea) at 50 W, 50 kHz, 0.7 mbar during 1 min, and then silanized with 2% of (3-Aminopropyl) triethoxysilane (Sigma) in Phosphate Buffer Saline (Sigma) followed by 0.5% of glutaraldehyde in PBS, each incubation lasting 30 min at room temperature. Between both incubations, the chip was washed three times in deionized water before immersing it in water, and incubating it at 4°C overnight. Two short silicone tubings were bonded in the connectors with epoxy glue (Sader). Then, five tungsten wires of 80 μm diameter (Goodfellow, United Kingdom) were first incubated during 30 min at room temperature in a 1% bovine serum albumin (BSA) solution in PBS, then gathered through the two silicone tubings before being positioned in the grooves of this COC base. Afterward, the COC plate was covered with a pressure sensitive PCR tape (ThermalSeal RTS^TM^, Excel Scientific, Sigma) except for the reservoir which stayed open (Serra et al., 2017). Finally, the central reservoir and the connectors were then incubated in degassed and filtered PBS for 1 h, at 37°C to absorbed bubbles forming at the grooves. Collagen I mix was prepared on ice by mixing collagen I from rat tail (Corning), PBS 10X, NaOH 1N and distilled water to obtain a final collagen concentration of 6 mg/ml at pH 7. As the collagen I mix is highly sensitive to temperature changes, the collagen I mix was continuously kept on ice at 4°C, and was gently blended with an appropriate spatula before being centrifuged less than 30 s to prevent air bubbles trapping and to pull them up, respectively. Afterward, the degassed PBS was removed from the COC plate beforehand put at 4°C during 15 min, and replaced with 80 μl of collagen I mix, gently poured above the open reservoir. The whole set-up was incubated at 37°C, 5% CO_2_ and under humidified atmosphere for 2 h, before placing a PBS droplet above the collagen I mix to keep it wet. This last step was crucial to shape empty tubes: the polymerized collagen I never stayed dry. Eventually, after collagen I polymerization, wires were gently removed through the connectors in order to get five empty cylindrical microchannels. From this step, the chip remained immersed in PBS or cell culture medium after the seeding.

### Coating and Cell Culture On-Chip

Once empty tubes in collagen I were shaped, they were covered with a thin layer of different proteins composing the ECM for 1 h, at 37°C, before cell seeding. Several proteins were individually investigated: collagen IV (Sigma), laminin (Sigma), and Matrigel (Corning; derived from the basal lamina secreted by a murine tumor, composed of many different proteins). To this end, laminin (Sigma) coupled or not with a fluorescent dye, rhodamin (Laminin-Rhodamin, Tebu-bio), was diluted in culture medium (0.02 mg/ml), as well as Matrigel (50:50). Collagen IV was mixed with water and NaOH 1N to reach a final concentration of 0.5 mg/ml. An acid acetic solution was used to adjust its pH to 7.4 (neutral pH). Afterward, 50 μl of those different coating proteins were slowly and gently manually injected with a P10 pipette in the collagen I tubes from a connector. The chip was then immersed in PBS, and incubated at 37°C, under humidified atmosphere, with 5% CO_2_ for 1 h. The channels were finally washed twice with PBS, and seeded.

Cells were concentrated at 5.10^6^ cells/ml in the appropriate culture medium. The MDCK cells were directly and carefully injected by pipetting within the tubes. However, they quickly passed through the tubes, decreasing the cell adhesion probability on the channel walls. Consequently, *Pkd1* cells that were smaller after the trypsinization step, were concentrated at 5.10^6^ cells/ml in differentiation medium mixed with 4% Dextran (70 kDa, Sigma). As for the coating proteins, cells were slowly and gently injected manually in the tubes, with a P10 pipette, to prevent air bubbles entry as much as possible with a liquid-liquid interface between the connector and the pipette tip. It was usually realized on both sides of the chip, through the two connectors. After the cell seeding, the chip was immersed in 8 ml of cell culture medium, in a Petri dish placed in the incubator, at 37°C, 5% CO_2_ atmosphere. Half of the medium was changed every 2 or 3 days and cells were followed during several weeks (generally more than 3 weeks).

### 3D Cell Labelling and Imaging

For live cells experiments lining the tubes, bright light images were acquired every 2–5 days with a cell culture microscope (Leica). For immunostaining experiments, chips were washed three times with PBS containing CaCl_2_ and MgCl_2_ (Sigma), then fixed with 4% paraformaldehyde for 15 min at room temperature: 3 ml of each solution were successively deposited on collagen I. During all the immunostaining protocol, the collagen I scaffold remained immersed in liquid to prevent it from drying. Carefully detached from the COC surface with thin tweezers, the collagen I scaffold was then immersed in a permeabilization buffer composed of 0.1% Triton X-100 (LifeTechnologies) and 2% BSA (Sigma) diluted in PBS for 5 min. The collagen I scaffold was then washed again three times with PBS to remove Triton X-100, and blocked in a solution of PBS with 4% BSA-0.1% Tween 20 for 2 h at room temperature, and under humidified atmosphere. For F-actin labeling, the tubular scaffold was labeled with phalloidin-TRITC (Sigma) and nuclei were counterstained with Hoechst (Sigma), in a humidified chamber and at room temperature for 45 min. Rinsed three times with the blocking buffer for 30 min each, the collagen I scaffold was then mounted with the VectaShield mounting medium (Vector Laboratories) in a homemade PDMS chamber, and imaged under a confocal microscope (Zeiss, PICT-IBiSA Imaging platform from Institut Curie).

### Image Representation and Analysis

Images acquired were analyzed on ImageJ software (NIH). For visual representation in figures, color balance was individually adjusted for each image. For some images in Figure 3, a denoising was performed with Safir ImageJ plugin (Kervrann and Boulanger, 2006).

For analysis of 3D confocal stacks at high resolution, *Pkd1*^+/-^ and *Pkd1*^-/-^ tube diameters in a horizontal section were manually measured at the center part of the field. Quantification of cell density was performed by manually counting nuclei on 100 × 50 μm^2^ area, randomly chosen for each image at the middle of the inferior half of the tube. Four images (over 28) with *Pkd1*^+/-^ tubes of aberrant sizes (superior to 125 μm), very likely corresponding to initial aberrant tubes, were removed from analysis. A home-written ImageJ macro was also developed in order to analyze confocal high-resolution images, and was used here to check shape modifications induced by *Pkd1*^-/-^ culture. Sequential steps were (1) enhancing of the local contrast of each image with the CLAHE plugin, (2) reducing the background noise, (3) fitting the external contour of the F-actin labeled tubes with an ellipse, producing an envelope of the tube and its transversal section over its length.

For F-actin orientation analysis in tubes, the ImageJ OrientationJ plugin was used (written by Daniel Sage at the Biomedical Image Group (BIG), EPFL, Switzerland)^1^ (Rezakhaniha et al., 2012). Briefly, for each pixel-centered window, the orientation is analyzed based on a structure tensor, and both an angle value (local predominant orientation) and a coherency value were obtained. Coherency is a measurement of the “strength” of the local orientation (coherency close to 1 for a strong local orientation, and to 0 for no preferential local orientation), and is defined as the ratio between the difference and the sum of the maximum and minimum tensor eigenvalues.

Images acquired with confocal at high resolution (40× objective) were rotated to yield horizontal tubes, and a maximum *z* projection of the inferior half of the tube (with the highest signal) was performed. The analysis was done on a rectangle corresponding to the center half of the projection (white rectangles in Figures 4A,D). This rectangle was drawn in the middle of the projection, where the effects of the curvature of the cylinder are minimal: we thus neglected this curvature in our analysis. “Distribution of orientation” menu was selected, giving a weighted histogram, with weight being the coherency. Histogram values presented were normalized by the surface area of the window (in pixels^2^). Following parameters were used: min-coherency = 0, local window σ corresponding to 2 μm (between 5 and 12 pixels depending on image zoom), gradient: cubic spline. A parallel analysis was performed with local windows of 8 μm with close results.

For the analysis of tubes along time: after acquisition of time movies of live cells experiments lining the tubes (Δt = 2–5 days, typical images in Supplementary Figure 2A), masks of cylindrical channel contours (Supplementary Figure 2B, left, day1 corresponding to Supplementary Figure 2A) and masks of regions filled with cells (example in Supplementary Figure 2B, right) were drawn using a drawing tablet (Cintiq, Wacom, Japan) and a home-written ImageJ macro. Superimposed images of mask-tubes (green) and mask cells (red) are shown in Supplementary Figure 2C: the yellow parts correspond to cells present in channels, the green parts to empty channel regions, the red parts above or under channel to cell “invasion” (protrusions or cells extending in collagen matrix). The analysis shown here is focused on the part of tubes that is common to all channels and all times (Supplementary Figure 2D, right): indeed, some chips encountered defects at left or right moieties of the chip along time, mainly due to cell growth from the groove region, that prevented the analysis of the corresponding part of the tube for these time-points. However, total individual channel data (Supplementary Figure 2D, left) were also collected, giving only marginal differences in the results.

In more details: first, for a given stack, horizontal contours were extracted from tube masks for diameter analysis (Supplementary Figure 2E), with the approximation that tubes were revolution surfaces and that the projection visible on images corresponded to local cylinder diameter. For cell density (Supplementary Figure 2F) and invasion (Supplementary Figure 2H) analysis, we determined the intersection between cell masks and tube masks (cell areas inside tubes), and cell masks with the exclusion of this intersection (cell invasion); global areas were calculated, as well as local cell densities (in function of *x*, principal direction of the tubes), defined as the sum of contributions of the different cell masks.

Data generated by the analysis of individual stacks were afterward aggregated for global statistics. Tubes with important deformations at initial times were excluded for the analysis (for the whole analysis, it concerned 25 out of 48 × 5 = 240 tubes, i.e., ∼10% of the tubes).

Binned cell densities as a function of time after seeding correspond to following sequential operations: 1. Mean of each tube local diameters along *x*; 2. For each time, mean on the selected tubes in each individual chip; 3. For each time bin (]0 3[ days, [3 6[ days), mean of the different values if the considered chip has several time points in the considered bin, 4. Mean and S.E.M. (Standard Error of the Mean) of the different chips were calculated (and represented at the upper limit of the binning interval). Correlations reported are the mean between tubes of the correlation coefficients (Matlab corcoeff) at each time, between adjacent external contours of two different tubes (tube correlation), or between the local diameter of one tube and the local spacing of the adjacent intertube. The kinetics of tube deformation were computed as a function of time after confluency, determined independently for each tube. For the study of maximal tube deformation over time, only tubes monitored for at least 6 days after confluency were selected, and we also checked that the results and the difference between conditions were similar considering a similar duration of observation after confluency.

Means and plots were performed on Kaleidagraph, Matlab and and Excel. Error bars refer to S.E.M. unless otherwise specified. *F*- and *t*-tests for statistical analysis were performed on Excel, assuming a normal distribution of the data.

## Results

### Reproducing Kidney Architecture on Chip

In order to investigate the formation of renal cysts and the likely associated snowball effect, the development of new *in vitro* models recapitulating the tightly packed organization of nephrons in the kidney is necessary. Here we focused on mimicking the geometrical and mechanical characteristics of parallel proximal tubules (the first segment of the nephron). We chose to develop a biomimetic scaffold with aligned, parallel and regularly spaced circular channels in a biocompatible and deformable hydrogel, in order to allow both mechanical and chemical coupling between tubes. Tube diameter should be as close as possible as *in vivo*, in the range of 50 μm for the lumen diameter (Knepper et al., 1977; Xi et al., 2017; Venzac et al., 2018). For that purpose, a microfabrication technique based on wire molding (Dolega et al., 2014; Weber et al., 2016; Venzac et al., 2018) was developed, in which collagen hydrogel was gelled around pre-positioned 80 μm diameter tungsten wires, followed by the removal of the wires to create parallel circular hollow channels in the hydrogel.

In practice, an open microfluidic chip was embossed on a cyclic olefin copolymer (COC) plate using a micro-milled brass mold. The structures consisted of an open rectangular reservoir to contain collagen I, with channels on each side containing five grooves each, sealed with a pressure sensitive PCR tape (Serra et al., 2017), in order to control the wire positions. We used horizontal connectors to silicone tubings to seed cells after collagen polymerization and wire removal (Figure 1). The production of a long-lasting mold with 3D features (including slopes and half cylindrical connectors) was only possible through micro-milling. Embossed COC allowed the reusability of the rigid microfluidic chip, and a good imaging due to its transparency, controlled thickness and low autofluorescence (Van Midwoud et al., 2012; Roy et al., 2013).

For hydrogel injection in the central chamber, we used collagen I, the main ECM component, at different concentrations: 2.5, 4, and 6 mg/ml. For 2.5 and 4 mg/ml, the circular channels were not stable and uniform, and some of them collapsed. Proper molding of five parallel channels was obtained with 6 mg/ml collagen, as previously reported (Weber et al., 2016), with a success rate of 90%. Diameters after demolding, and immediately after cell seeding (D_0_), were compared to the initial diameter of the 80 μm tungsten wire: we observed an increase of diameter, as assessed by its horizontal projection, of about 20% of the expected diameter after the different microfabrication steps, principally due to the demolding step (Supplementary Figure 1A). The first experiments were performed with a distance of 200 μm between the cylinders, which was afterward reduced to 100 μm to obtain more tightly packed tubes.

These cylindrical channels were then coated or not with different proteins constituting the basal membrane, which is mainly composed of laminin and collagen IV (Rahilly et al., 1991; Miner, 1999; Ogawa et al., 1999): both laminin and Matrigel were used, with efficient coating assessed with fluorescent laminin-Rhodamin (Supplementary Figure 1B). To evaluate the potential of this device to reproduce nephron structures, different renal cell lines were used to create kidney tubes: MDCK cells, and cells derived from mouse proximal tubule and models for ADPKD (Supplementary Figure 5, *Pkd1*^-/-^ and *Pkd1*^+/-^). Cells were seeded from the horizontal inlet with a density of 5.10^6^ cells/ml. The initial density after cell adhesion in tubes was difficult to control, so that in order to limit any related bias, the kinetics of each tube deformation was analyzed taking tube confluency at the starting point. The cells were then kept in culture during up to 2 months, cell colonization and tube deformation were monitored and analyzed over time.

### Control MDCK Cells Colonize Tubes and Do Not Lead to Tube Dilation

**FIGURE 2:**
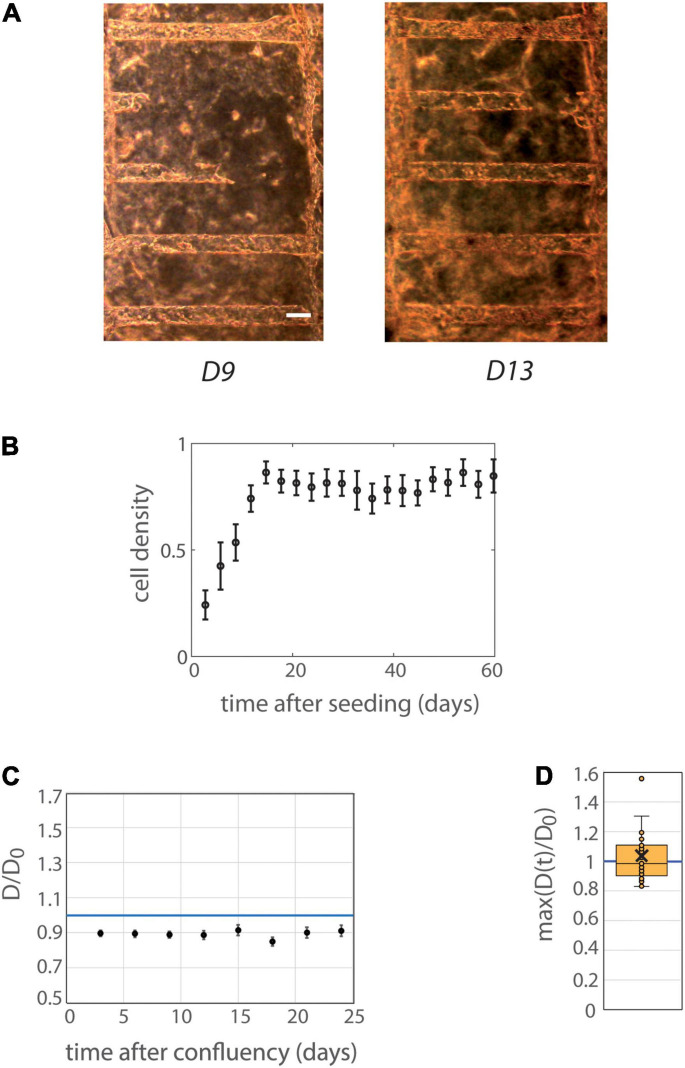
Behavior of MDCK cells in tubes. **(A)** MDCK-Lifeact-GFP cells were seeded within tubes molded in collagen I at 6 mg/ml, and observed under an optical microscope over time. The temporal evolution at days 9 and 13 after seeding is represented (before and at confluency) is represented. Scale bar: 100 μm. **(B)** Mean cell density over time (*n* = 12 MDCK chips). The temporal scale refers to time after cell seeding for colonization curves. Tubes with or without laminin coating were pooled, because of similar behaviors in these two conditions (see Supplementary Figure 8 for separated behavior). The mean curve remains below cell density 1 (corresponding to full colonization) reflecting the fact that some tubes were never fully colonized with cells. Error bars: S.E.M. **(C)** Kinetic evolution of mean tubes diameter normalized by diameters at seeding as function of the time after tube confluency. The temporal scale refers to time after confluency for curves of tube deformation. A blue horizontal line at D/D_0_ = 1, corresponding to no change in diameter, is indicated. Chips with or without laminin coating were pooled. Each time point corresponds to 19–30 tubes. Error bars: S.E.M. **(D)** Maximum (over time) of the mean normalized diameter. Tubes with or without laminin coating were pooled. Points correspond to individual tube values. Central bar, median; cross, mean; box, values between Q1 and Q3 quartiles; error bars, extreme values [between Q1 - 1.5*(Q3 - Q1) and Q3 + 1.5*(Q3 - Q1)]. **(C,D)** were computed only for tubes having reached full confluency during the observation period.

MDCK cells were first used as a classical model of renal cells (de Beco et al., 2009; Delous et al., 2009; Reffay et al., 2014; Bhoonderowa et al., 2016) for initial tests on cell viability, colonization and mechanical deformations of collagen-based tubes. MDCK cells were able to colonize nicely tubes, and to survive 1–2 months in tubes (Figure 2). Cells in tubes were organized in monolayer, and reached confluency with cohesive intercellular junctions, as assessed by ZO1 (Zonula occludens-1) labeling of tight junctions (Supplementary Figures 3A,B). Tubes exhibited no or minimal dilation over time, up to two months. We did not observe any significant influence of the coating on the colonization time or tube diameters (Supplementary Figure 3C). Mean data binned with 3-days interval are presented in Figures 2B,C. Confluency was achieved in ∼2 weeks after cell seeding (Figure 2B). A mild decrease of mean tube diameters (normalized with diameter at seeding, Figure 2C) occurred at early time points even before confluency, with an amplitude of ∼10% of the initial diameter. The tube diameter remained thereafter constant after confluency, for about 1 month (Figure 2C). Figure 2D depicts the maximum over time of the mean diameter of all different individual tubes. It was very close to 1 (1.03 ± 0.04, *n* = 31), further illustrating the absence of tube dilation after seeding with cells.

Altogether, these results show that MDCK cells colonized efficiently tubes, without dilating the tubes along time.

### Organization of PCT *Pkd1*^-/-^ and *Pkd1*^+/-^ Cells in Tubes

In order to assess the specific mechanical behaviors of cells model for ADPKD in this biomimetic multitubular device, PCT *Pkd1*^-/-^ and *Pkd1*^+/-^ cells were seeded in tubes. These isogenic cell lines were derived from proximal tubule cells of a transgenic *Pkd1*^*flox/–*^ mice; the resulting cell line was transfected or not with Cre recombinase to yield *Pkd1*^-/-^ cells and *Pkd1*^*flox/–*^ cells that function effectively as *Pkd1*^+/-^ cell lines (Shibazaki et al., 2008). The PCT *Pkd1*^-/-^ cells were previously characterized as an ADPKD model, forming cysts when cultured in a 3D collagen/Matrigel matrix, while the control *Pkd1*^+/-^ cells self-organized in tubules instead (Wei et al., 2008). Concerning the choice of a proximal cell line, it is important to note that ADPKD cysts have been observed in all parts of the nephron (proximal and distal) (Baert, 1978; Torres and Harris, 2006; Vujic et al., 2010). In human models, a contribution of proximal cysts was observed from aquaporin immunolabeling and early microdissection studies (Huseman et al., 1980; Bachinsky et al., 1995; Hayashi et al., 1997), while in the different animal models the situation appears heterogeneous, with studies suggesting cysts originate from the collecting tubes before extending to the different segments (Hopp et al., 2012; Saito et al., 2018), but with possible underestimation of the proximal contribution due to differentiation issues (Hopp et al., 2012), and high sensitivity to initial conditions (Leonhard et al., 2016). In our experiments, while heterozygous *Pkd1*^+/-^ cells mostly behaved like WT PCT cells in 2D or 3D (not shown), homozygous PCT *Pkd1*^-/-^ cells, lacking the functional *Pkd1* gene on both chromosomes, exhibited hallmarks characteristic for ADPKD, including an increased proliferation rate (∼1.8-fold, Supplementary Figure 4) (Wei et al., 2008) and an increased extrusion in confluent cultures (not shown). Basic properties of adhesion and apico-basal polarity were assessed by RT-qPCR on cells cultured in 2D. We observed no significant change in the expression of actin or cell-matrix adhesion genes, but a significant decreased expression for apico-basal polarity markers ezrin and Na/K-ATPase, and for intercellular adhesion E-cadherin and N-cadherin genes, in PCT *Pkd1*^-/-^ compared to *Pkd1*^+/-^ cells (Supplementary Figure 5).

**FIGURE 3:**
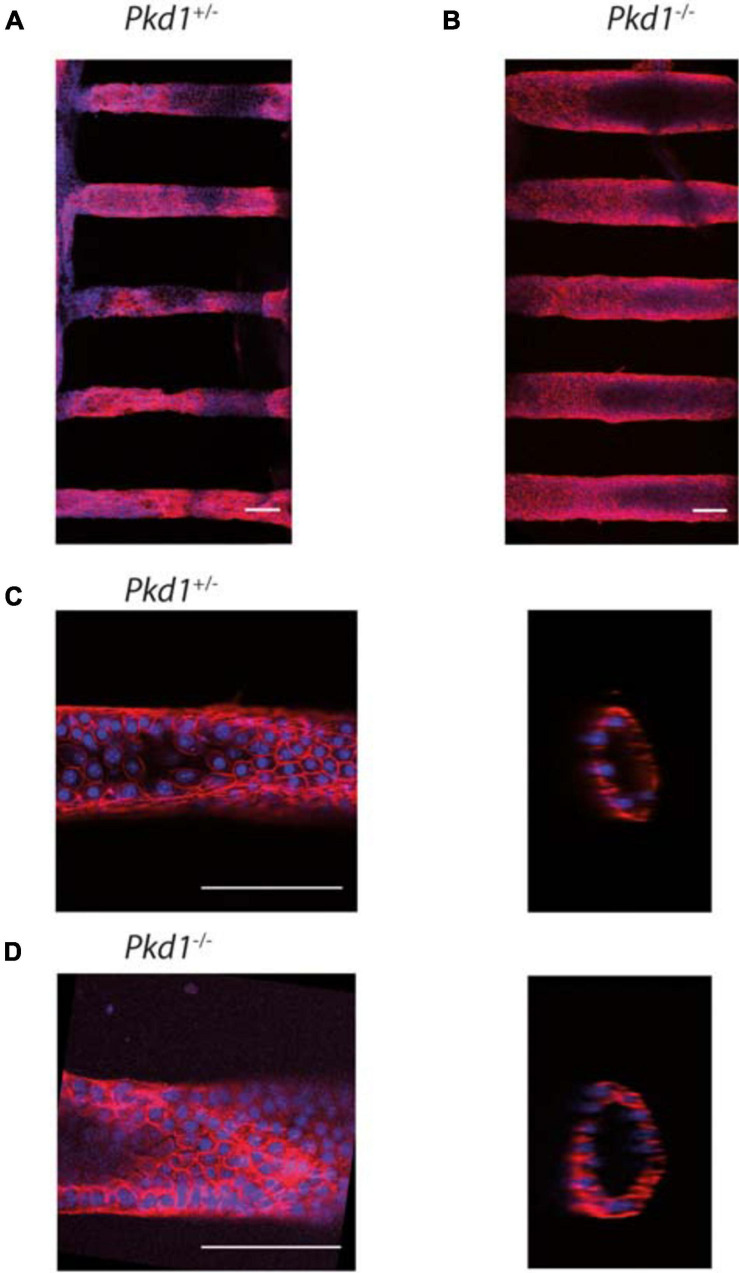
PCT *Pkd1*^+/-^ and *Pkd1*^-/-^ organization in tubes. Cells were labeled for F-actin and nuclei, and imaged at confocal both at low and high resolution to study the global cell organization and F-actin organization. Confocal images of tubes labeled with phalloidin-TRITC (red) and Hoechst (blue). **(A,B)** global organization of *Pkd1*^+/-^ **(A)** and *Pkd1*^-/-^ **(B)** tubes imaged at a low resolution (10× objective). Maximal *z* projections. Mean tube diameters of tubes in the chips shown, as assessed by *z* projection, were, respectively, ∼85 and 135 μm for the chips shown in **(A,B)**, in agreement with *Pkd1*^-/-^ tube deformation. **(C,D)** *Pkd1*^+/-^ **(C)** and *Pkd1*^-/-^ **(D)** tubes imaged at a high resolution (40× objective) in steps corresponding to the first early steps of tube dilation for Pkd1^-/-^ cells. Confocal section (left) and orthogonal projection (right). A median filter (3 pixels) was applied on orthogonal projection images. Scale bar, 100 μm. Mean diameters measured for tubes imaged at high resolution were 85 ± 10 μm for the *Pkd1*^+/-^ condition and 95 ± 5 μm for the *Pkd1*^-/-^ condition for high resolution images (S.D. are indicated). Here 24 images of *Pkd1*^+/-^ tubes and 10 images of *Pkd1*^-/-^ tubes were done (performed, respectively, on 8 and 7 chips).

To promote efficient tube colonization, cells were seeded in tubes in proliferative state, and differentiation was initiated right after seeding. All PCT cell lines colonized the tubes and could be kept in culture up to 1 month. The prominent feature was that *Pkd1*^-/-^ cells dilated tubes over time, whereas control *Pkd1*^+/-^ cells did not. Before describing how renal cells could affect tube structure, we first present their global organization in the 3D collagen scaffold (Figure 3). Cell monolayers with lumen formation were observed a few days after confluency for both cell lines. At this early stage, *Pkd1*^-/-^ tubes already appeared more dilated and circular than *Pkd1*^+/-^ tubes (Figures 3C,D). We also observed an increased cell density in *Pkd1*^-/-^ tubes compared to *Pkd1*^+/-^, respectively, 94 ± 7 cells/10 000 μm^2^ (*n* = 10), vs. 53 ± 3 cells/10 000 μm^2^ (*n* = 22; p < 10^–5^), in line with the increased proliferation for the ADPKD model already reported in 2D.

**FIGURE 4:**
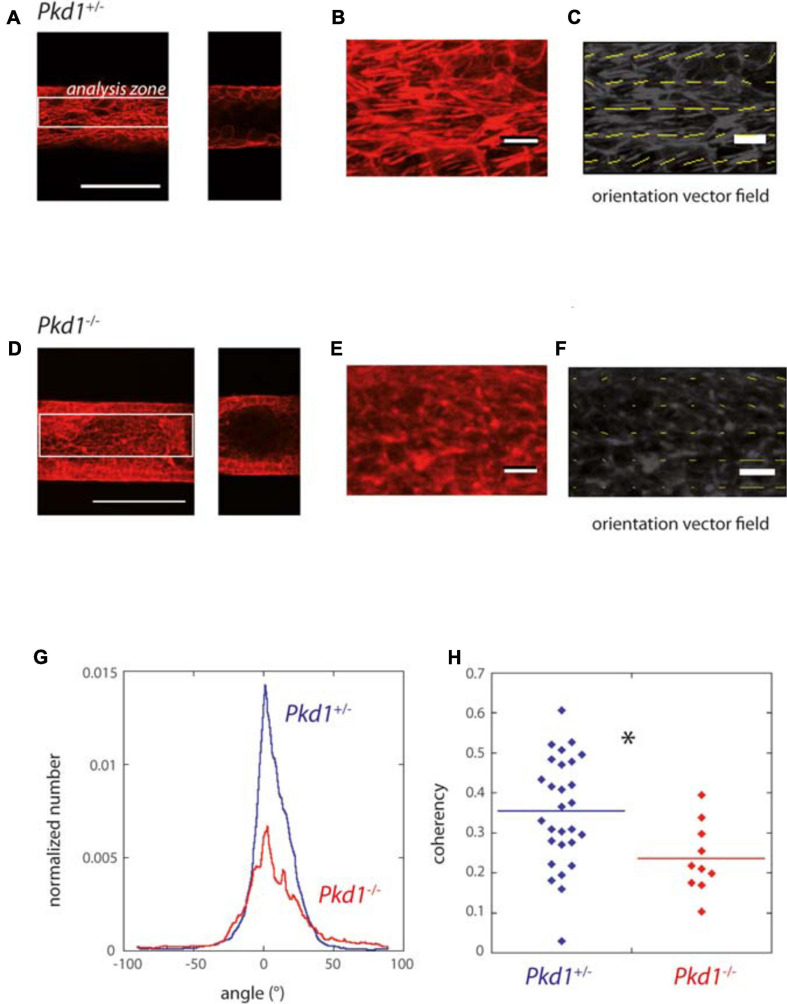
F-actin orientation of PCT *Pkd1*^+/-^ and *Pkd1*^-/-^ cells in tubes. **(A–F)** F-actin labeling in *Pkd1*^+/-^ **(A–C)** and *Pkd1*^-/-^ **(D–F)** tubes. **(A,D)** Left: *z* projection of the inferior half of the tube is shown, scale bar 100 μm. Right, confocal section at the middle of the tube. OrientationJ analysis was performed in a central rectangle corresponding to half of the tube (white rectangle in **A,D**), in order to get rid of border effects. **(B,E)** Zoomed part, **(C,F)** Orientation vector fields (yellow arrows). Magnitude normalized by the strength of orientation (coherency) is represented. Coherency is low in the *Pkd1*^-/-^ condition, so that arrows are barely visible in **(F)**. **(G)** Distribution of F-actin local orientation as assessed by OrientationJ software for PCT *Pkd1*^+/-^ (blue) and *Pkd1*^-/-^ (red) cells. The analysis was done at a subcellular scale, with a 2 μm local analysis window. Histograms given by OrientationJ are pondered by coherency, meaning that the angle determined for a given window has a more important contribution if there is a clear-cut local orientation. Each histogram is normalized by the size of the analyzed area (in pixels^2^) before averaging. The analysis was performed on pooled coating conditions (laminin, ECM and collagen), with the majority of tubes corresponding to laminin coating in *Pkd1*^-/-^ and *Pkd*^+/-^ conditions. **(H)** Mean coherency (per pixel) for PCT *Pkd1*^+/-^ (blue) and *Pkd1*^-/-^ (red) cells. ^∗^Statistically significant difference with *p* < 0.05. Each point corresponds to one image.

ADPKD is associated with disorders in cellular orientation, in particular misaligned divisions and loss of planar polarity (Fischer et al., 2006; Happe et al., 2011a; Nigro et al., 2015). The orientation of the F-actin fibers, reflecting cytoskeleton organization and cell orientation, was specifically assessed in our system. We observed in most cases numerous stress fibers, mostly aligned along the *Pkd1*^+/-^ tube axis, while *Pkd1*^-/-^ tubes exhibited either a similar pattern or more disorganized fibers. A quantification was performed by OrientationJ analysis of the *z* projection of the lower half of tubes, a global measurement which included primarily stress fibers in the basal plane, but also the contour of cells in the middle plane (Figures 4A–F). Both *Pkd1*^+/-^ tubes and *Pkd1*^-/-^ tubes exhibited a clear F-actin alignment along the tube axis (Figure 4G), with about half of angles ranging between −10° and 10° for both cell lines (55% for *Pkd1*^+/-^ tubes and 42% for *Pkd1*^-/-^). An important difference between the two conditions is that the density of oriented fibers appeared higher in the *Pkd1*^+/-^ condition (Figures 4A–F). This was quantified by a coherency measurement (Rezakhaniha et al., 2012; Clemons et al., 2018), where coherency is a measurement of the strength of orientation, close to 1 for a strong local orientation, and to 0 for no preferential local orientation. Figure 4H shows a statistically significant difference between the coherency in *Pkd1*^+/-^ and *Pkd1*^-/-^ tubes (p < 0.05, Figure 4C, respective coherency values 0.35 ± 0.03, *n* = 28, and 0.23 ± 0.03, *n* = 10). This illustrates a denser organization of parallel F-actin fibers oriented along the tube axis for *Pkd1*^+/-^ cells. This denser array of F-actin may be linked to two phenomena: the orientation of the cell division axis (not quantified), and the mechanical stabilization of the soft tube. This observation is in agreement with a model where dividing *Pkd1*^+/-^ cells would tend to push cells in the direction of tube elongation, and not to dilate tubes, contrary to dividing *Pkd1*^-/-^ cells.

In conclusion, shortly after confluence, cells were organized in monolayers in the 3D circular collagen scaffold in the different coating conditions. *Pkd1*^-/-^ tubes, slightly dilated even for short culture time and exhibited an increased cell density, and a decreased density of F-actin fibers oriented along the tube axis.

### PCT *Pkd1*^-/-^ Cells Lead to Strong Tube Dilation, Contrary to Their Isogenic Control

**FIGURE 5:**
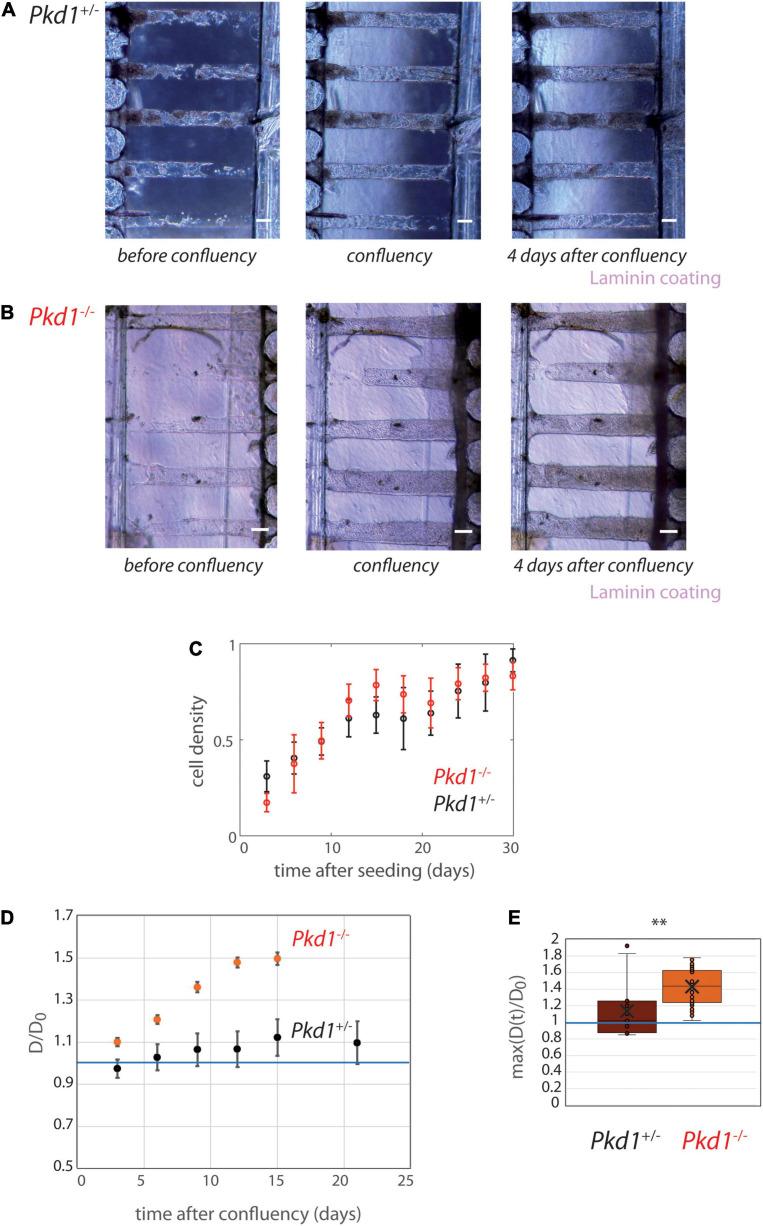
PCT *Pkd1*^+/-^ and *Pkd1*^-/-^ tube deformation in chips with 200 μm spacing. **(A,B)** Examples of temporal evolution of tubes with laminin coating, for *Pkd1*^+/-^ cells **(A)** and *Pkd1*^-/-^ cells **(B)**. Scale bar:100 μm. Days after seeding: **(A)** 11, 16 (confluency), 20, **(B)** 9, 10 (confluency), 14. **(C–E)** Quantitative analysis, *n* = 12 *Pkd1*^+/-^ chips (black) and *n* = 14 *Pkd1*^-/-^ chips (red), all coatings pooled (see Supplementary Figure 8 for separated behavior). **(C)** Mean cell density over time. Error bars: S.E.M. **(D)** Kinetic evolution of mean tubes diameter normalized by diameters at seeding, in function of the time after tube confluency. A blue horizontal line at D/D_0_ = 1, corresponding to no change in diameter, is indicated. Each time point corresponds to 8–35 tubes for *Pkd1*^-/-^, 9–20 tubes for *Pkd1*^+/-^. Error bars: S.E.M. **(E)** Maximum (over time) of the mean normalized diameter. Points correspond to individual tube values. Central bar, median; cross, mean; box, values between Q1 and Q3 quartiles; error bars, extreme values [between Q1 - 1.5*(Q3 - Q1) and Q3 + 1.5*(Q3 - Q1)]. **(D,E)** were computed only for tubes having reached full confluency during the observation period. ^∗∗^ indicates statistically significant difference with *p* = 0.0002.

The behavior of PCT *Pkd1*^-/-^ and *Pkd1*^+/-^ cells lining collagen tubes after confluency was further investigated as a function of time (Figure 5 and Supplementary Figures 7–9). Both cell types colonized efficiently the tubes in 10–15 days, with a colonization rate that seemed quicker for *Pkd1*^-/-^ cells (Figure 5C). As already observed right after confluency and as expected for non-ADPKD conditions, no significant dilation of the tubes was observed for *Pkd1*^+/-^ tubes, regardless of the coating (Figure 5A and Supplementary Figures 7, 8). In particular, D/D_0_ remained close to 1 over time (Figure 5D), as well as the maximum over time of the normalized diameters (1.13 ± 0.09, *n* = 15, Figure 5E). Short cytoplasmic extensions into the collagen were rarely observed for both cell types (Supplementary Figures 7A, 10). At last, first results on PCT WT cells suggested that they behaved similarly to PCT *Pkd1*^+/-^ cells, with no mean tubular dilation (Supplementary Figure 6).

On contrary, tube dilation was consistently observed in *Pkd1*^-/-^ tubes independently of coating conditions (Figure 5B and Supplementary Figures 7, 8). The kinetic evolution illustrates a mean *Pkd1*^-/-^ dilation of ∼60% compared to the initial value (Figure 5D). The maximum deformation (over time) was 1.43 ± 0.03 (n_*tubes*_ = 28) reflecting a large tube dilation (Figure 5E). Tubes remained globally homogeneous in diameter when dilated (Supplementary Figure 9A). Altogether, these experiments showed that *Pkd1*^-/-^ lining collagen tubes induce a significant tube dilation.

Once demonstrated that *Pkd1*^-/-^ cells induced a significant tube dilation, we investigated whether the five tubes present in collagen were mechanically coupled. To do so, we performed several quantifications. The intertube spacing was measured and showed a decrease from 190 μm to ∼160 μm 25 days after seeding (Supplementary Figure 9B). This intertube spacing was compared to the local tube deformation: a clear anticorrelation was observed (Supplementary Figure 9C), in agreement with the idea that the tube deformation led to a short-scale remodeling of the intertube matrix. This suggested that each dilation event was independent. At last, correlation coefficients were calculated between adjacent contour lines in adjacent tubes (Supplementary Figure 9D), in order to evaluate if local deformations of one tube spatially corresponded to local deformations of the neighbor tube. These data showed that, in these conditions (tube spacing of 200 μm), tube dilations were not significantly coupled.

### *Pkd1*^-/-^ Tubes Come in Contact After Dilation in 100 μm Spaced Chips

**FIGURE 6:**
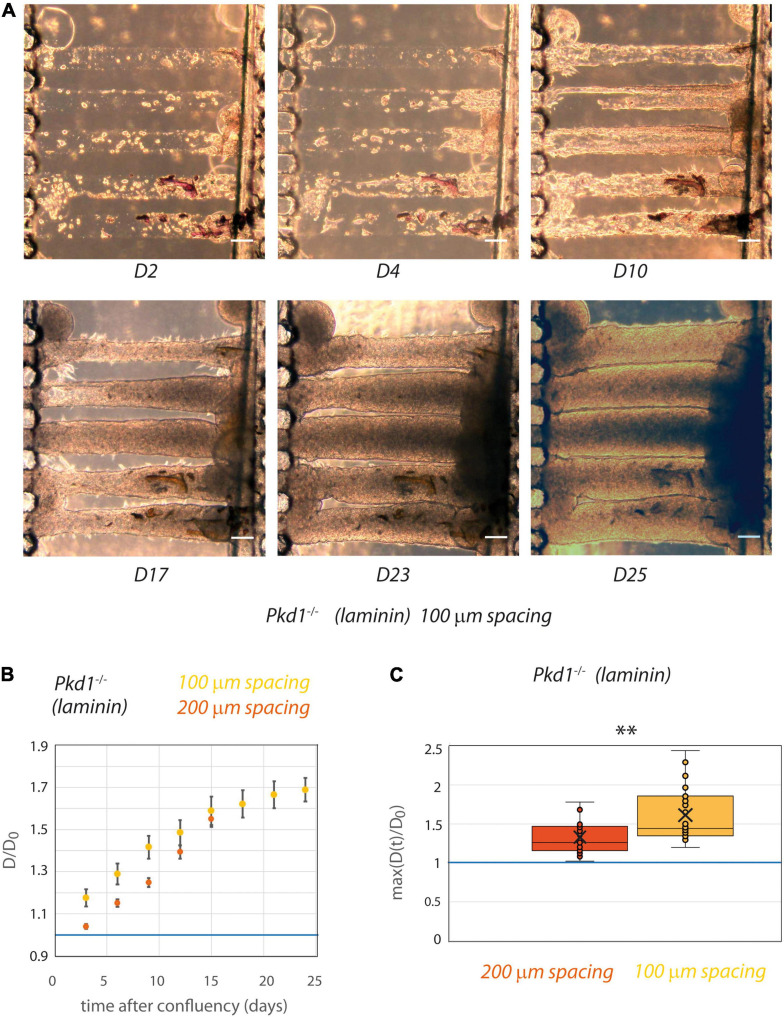
PCT *Pkd1*^-/-^ tube deformation in chips with 100 μm spacing. **(A)** Example of temporal evolution of tubes seeded with *Pkd1*^-/-^ cells at days 2, 4, 10, 17, 23, and 25 after seeding. Scale bar:100 μm. **(B,C)** The behavior in 100 μm spacing tubes (yellow) was assessed with laminin coating and compared to the behavior in 200 μm spacing laminin-coated tubes (red). **(B)** Kinetic evolution of mean tube diameter normalized by diameter at seeding, in function of the time after tube confluency. A blue horizontal line at D/D_0_ = 1, corresponding to no change in diameter, is indicated. Each time point corresponds to 9–26 tubes for 100 μm spacing, 4–19 tubes for 200 μm spacing. Error bars: S.E.M. **(C)** Maximum (over time) of the mean normalized diameter. Points correspond to individual tube values. Central bar, median; cross, mean; box, values between Q1 and Q3 quartiles; error bars, extreme values [between Q1 - 1.5*(Q3 - Q1) and Q3 + 1.5*(Q3 - Q1)]. **(B,C)** were computed only for tubes having reached full confluency during the observation period. ** indicates statistically significant difference with *p* = 0.001.

Although the matrix stiffness allowed the tubes to be mechanically deformed in an ADPKD model, a spacing of 200 μm seems to be too important for the propagation of a mechanical coupling. The spacing between tubes was thus reduced to 100 μm after optimization of the micro-milling technique (Figure 6). Similar experiments with laminin coating only were conducted with tube spacing of 100 μm. As a control, we verified that even in this close proximity *Pkd1*^+/-^ cells still did not deform collagen tubes (not shown). Strikingly, with this reduced spacing between tubes, some *Pkd1*^-/-^ tubes could be in close proximity after dilation, with the creation of a plane interface between tubes (Figure 6A). We observed that both the rate of deformation and the maximal deformation induced by *Pkd1*^-/-^ cells were increased in the 100 μm spaced tubes compared to the 200 μm spaced condition (Figures 6B,C). The maximum over time of the normalized diameters in 100 μm spacing chips was significantly larger than in the corresponding laminin-coated 200 μm spacing chips (respectively, 1.6 ± 0.07, *n* = 26, vs. 1.33 ± 0.06 for laminin-coated conditions, *n* = 15, *p* = 0.001, Figure 6C). These data strongly suggest that tube proximity modulates the geometry and the rate between neighbor tube deformation in an ADPKD model.

## Discussion

In this paper, we describe the development of a new generation of kidney-on-chip with parallel aligned circular tubes, of 80 μm diameter and 100 or 200 μm spacing, in a biocompatible and deformable collagen I. This chip was designed in order to reproduce geometrical, mechanical and biological characteristics of an array of renal proximal tubules with the aim to study physiopathological mechanisms of ADPKD. We first observed that our tubes were nicely colonized by different renal cells, with a long-term survival, in agreement with literature (Weber et al., 2016). No mean tubule dilation was observed with MDCK tubes. It is noteworthy to mention that MDCK tube dilation might have been triggered with some drug treatments, like cAMP agonists, as reported for renal cells in a bioengineered guided kidney tubule array system, where forskolin treatment of mIMCD3 cells induced a transformation from tubules to progressively dilating cystic structures (Subramanian et al., 2018). However, the scope of our study was to study the behavior of specific ADPKD models. Then we showed that contrary to *Pkd1*^+/-^ cells, *Pkd1*^-/-^ cells, as an ADPKD model, were able to induce an important tube deformation. These observations are in agreement with tubular dilation expected for this disease demonstrating the physiological relevance of our model. Furthermore, our multitube chip design with spacing between tubes reduced to 100 μm allowed for the first time to reveal possible mechanical coupling between tubes, which could play a central role in ADPKD cyst propagation.

The behavior of proximal tubular cells has recently been described in single tubes in collagen I (120 μm diameter) with collagen IV coating (Weber et al., 2016). The authors showed that in this system, proximal tubular cells were able to recapitulate most of their physiological functions. In our study, we extended the potential of such approach by recapitulating for the first time the close proximity observed between parallel adjacent nephrons on chip, and we specifically focused on an ADPKD cellular model.

*In vivo*, the spacing between nephrotic tubes is heterogeneous, and no mean value between nearest neighbors could be extracted from the literature. However, *in vivo*, tubes are mostly in closer contacts (tens of μm). The initial choice of 200 μm spacing was mainly imposed by ease of microfabrication. However, to further investigate the possible coupling between tubes we also push the microfabrication limits to reduce the spacing to 100 μm. Importantly, the latter spacing was small enough so that ADPKD tubes could be in direct contact after dilation, thus opening the way to a study of mechanical coupling between tubes.

Regarding the implementation of kidney features on chip, the collagen scaffold stiffness (around 1 kPa; Verhulsel et al., 2016) was in the same order of magnitude, albeit a little lower, as the kidney stiffness measured by elastography techniques (4–10 kPa, or higher in some pathologies; Derieppe et al., 2012; Moon et al., 2015; Samir et al., 2015; Hassan et al., 2016; Liu et al., 2017). However, these elastography-derived values are global values for kidney, and not local values.

Moreover, the application of a physiological flow within the tube will be central in future implementations of our chips, as tubular cells mediate flow information by mechanotransduction pathways (including primary cilia) for the organization of architecture. Technological challenges result here from the common input between tubes due to their very close proximity, and resulting in inhomogeneities in flow values because of different diameters or obstructions. Flow might also be useful to prevent possible cell aggregation in tubes that might occur in late stages, although our data suggest that at least initial tube deformation occurred for cells monolayers. Indeed, 3D organization in the whole time course of deformation remains to be studied, and may include transient events of multilayering or fillling that will be addressed in further studies. Concerning ECM composition, the basal ECM *in vivo* consists mainly in laminin and collagen IV isoforms (Miner, 1999). These coatings were reproduced on chip, but we did not observed any significant difference of the global behaviors with laminin coating compared to the situation of collagen I without coating (Zhang et al., 2009); a slight increase in *Pkd1*^-/-^ tube dilation was observed in uncoated conditions, but would need further confirmation. In addition, the coating may have an influence on subtler cellular behaviors within tubes, that were not analyzed here in details. A weak influence of the coating on the parameters assessed may be due to a degradation by cells, or to the secretion of their own extracellular matrix, possibly coupled to a lack of stability of the coating before full colonization (which could last 1–2 weeks).

Finally, our chip allowed to reproduce tube dilation observed for ADPKD tubes. In our configuration where we seeded a homogeneous *Pkd1*^-/-^ population, we expect a rather homogeneous tube dilation, which was indeed observed here. In the disease, primary cyst formation results from a local tube dilation, which is believed to be due to a somatic second-hit mutation (Cornec-Le Gall et al., 2014) generating heterogeneous cell populations in one tube; this more complex configuration was not studied here. Several causes, including proliferation and altered planar polarity, are known to be involved in ADPKD cyst formation (Nadasdy et al., 1995; Fischer et al., 2006; Castelli et al., 2013). Both factors were seen in our *in vitro* tubes, and in particular F-actin orientation could be assessed in a geometrically and mechanically relevant controlled environment. We observed that both *Pkd1*^+/-^ and *Pkd1*^-/-^ cells were able to sense tube curvature and to generate F-actin stress fibers aligned with tube direction. However, the density of oriented F-actin fibers was significantly higher for *Pkd1*^+/-^ cells. First, the orientation of division axis may be related to F-actin dynamic organization imposed by the geometry of the substrate, as described in other systems (Théry et al., 2005, 2007; Fink et al., 2011). In that case, dividing *Pkd1*^+/-^ cells would tend to push cells in the direction of tube elongation, and not to dilate tubes. Second, a dense array of parallel F-actin fibers may provide a mechanical consolidation for the shape of the tube, still helping to prevent its deformation.

The strong tube dilation observed with *Pkd1*^-/-^ cells led to tubes coming in very close contact after dilation, in the chips with 100 μm spaced tubes. When in close contact through a thin deformable substrate, tubes were mechanically coupled as expected, as also revealed by the linear shape of the created interface. But even before the complete contact was reached, tubes deformed more, and at a higher rate, than tubes separated with 200 μm, highly suggesting that an interaction at distance already occurred at this stage. This behavior could be due to mechanical or chemical effects, or a combination or both. Although the determination of the mechanisms involved in cooperative tube dilation is beyond the scope of this paper, some possible mechanisms are discussed here. First, chemical communication, which can occur at small distances inferior to 200 μm (Gerecht et al., 2010), could be involved in communication between renal tubes (El-Achkar and Dagher, 2015), with possible release of signaling molecules promoting proliferation or cyst growth (Kenter et al., 2019), including cytokines that may be released by epithelial cells upon mechanical stimulation (Kishikawa et al., 2002; Yamamoto et al., 2002; Wu et al., 2017). Second, mechanical mechanisms could include both cell reactions to mechanical stimuli by mechanotransduction pathways, and physical effects linked to the thinning of the ECM layer between adjacent tubes. Mechanotransduction events triggered by mechanical stimuli may include the YAP pathway, and it will be important to study how the impaired mechanotransduction by polycystins, including the RhoA-YAP-c-Myc axis (Happe et al., 2011b; Cai et al., 2018), is related to the tube deformation observed. Additive physical mechanisms may be related to ECM characteristics, with on one side possible easier ECM thinning for very thin layers between two tubes (Shull and Creton, 2004); on the other side a possible weakening of the ECM due to cell protrusions, in line with a possible metalloproteinase involvement in ADPKD (Obermüller et al., 2001; Tan and Liu, 2012).

## Conclusion

In conclusion, the strengths of our approach are the recapitulation of arrays of tightly packed deformable proximal tubes, and its application to an ADPKD model, with tube dilations and cooperative deformations. In the future, our study may also advantageously be extended to the study of human ADPKD models. While the reconstitution of the complete interactions involved in tubular cell behavior is beyond the scope of this study, the current limitations of our system include the absence of surrounding structures, like the peritubular capillary network. They also include the absence of a continuous physiological flow, and possible events of multilayering at middle or late stages of tube dilation.

Future studies will aim to distinguish mechanical and chemical contributions by applying pure mechanical stimulations on tubes, and to analyze matrix digestions or other possible mechanical modifications. Altogether, the cross-talk between renal tubes in our multitube chip is in good agreement with the cooperative behavior of snowball effect involved in cyst propagation in ADPKD. In line with these results, our device may also be of interest to screen for drugs that would inhibit this cross-talk behavior favoring renal cyst propagation.

## Data Availability Statement

The original contributions presented in the study are included in the article/Supplementary Material, further inquiries can be directed to the corresponding author/s.

## Author Contributions

SC, SDs, and SDm conceived the study. SM, BV, and BL performed the majority of the experiments. GG participated to the microfabrication and microfluidic experiments. FC and AB were involved in the cell culture and cell characterization. BC, CC, and IB participated in the image analysis. SC wrote the manuscript with input from all authors. SC and SDs supervised the project. All authors contributed to the article and approved the submitted version.

## Conflict of Interest

The authors declare that the research was conducted in the absence of any commercial or financial relationships that could be construed as a potential conflict of interest.

## Footnotes

1 http://bigwww.epfl.ch/demo/orientation/
