# Supplementary material for: A Multitubular Kidney-on-Chip to Decipher Pathophysiological Mechanisms in Renal Cystic Diseases
Source: Front Bioeng Biotechnol. 2021 May 26;9:624553. doi: 10.3389/fbioe.2021.624553 (PMC8188354; doi:10.3389/fbioe.2021.624553)
Supplement: Supplementary file 1 [file Data_Sheet_1.DOCX]

**A multitubular kidney-on-chip**

**to decipher pathophysiological mechanisms in renal cystic diseases**

Myram et al.

**Supplemental Information**

**1. Additional technological information**

We give here specifications about tube dimensions measured at different steps of the microfabrication process and visualization of laminin coating.

***Sup. Fig. 1:***

**(A)** Comparison of tube diameters (horizontal projection) during molding (D_wire_), after demolding (D_demolding_) and immediately after cell seeding (D_0_). D_0_/ D_wire_=1.22±0.13, n=12, D_0_/ D_demolding_=1.07±0.08, n=31. Error bars are S.D. **(B)** Laminin coating efficiency as visualized by laminin-Rhodamin in tubes.

**2. Additional description for analysis in tubes**

Data analysis method detailed in the Materials and Methods part is illustrated here.

***Sup. Fig. 2:***

On images at low resolution **(A)**, masks for cells and tubes were manually drawn **(B)**. Their superimposition in **(C)** allowed us to define different regions: green, part of the tubes not filled with cells, yellow, part of the tubes filled with cells, red, parts of cells outside the tubes (only the red parts around tubes, and not the red parts on sides, were considered for further analysis and corresponded to “invasive” behaviours). **(D)** An additional complexity was observed, because all tubes did not have the same *x* origin if some defects (e.g. bubbles) were initially present at the side (see tube 2, time1 contours); and defects due to cell growth from the side could expand over time, limitating the part of tubes that could be analyzed. In order to be able to perform spatial and temporal correlations over time, we selected only the part of the chip that could be visualized for all times and all tubes (this correspond to “l_alltubes,alltimes_” on the right). This was compared with a parallel analysis with the whole tube data at each time for the parameters were no correlation was needed (like mean diameters), with similar results. **(E)** First, for a given stack, horizontal contours were extracted from tube masks for diameter analysis. **(E-G)** Local diameters in function of the position (left) and mean diameters (right) were computed, as well as **(F)** the local cell density (between 0 and 1 at each position) and **(G)** the local distance between two adjacent tubes. For cell density **(F)** and invasion **(H)** analysis, we determined the intersection between cell masks and tube masks (cell areas inside tubes), and cell masks with the exclusion of this intersection (cell invasion); global areas were calculated, as well as local cell densities (in function of the position *x*), defined as the sum of contributions of the different cell masks.

**3. Additional data for MDCK cells**

We show here some confocal images of MDCK organization in tubes.

***Sup. Fig. 3:***

**(A)** Images representing the MDCK-Lifeact-GFP actin cytoskeleton organization, lining tubes molded in collagen I at 6 mg/ml, and observed under a confocal microscope at D4 post-seeding. Left, general view on three tubes; right, magnification on the second tube. Scale bar: 100 µm (left), 50 µm (right). **(B)** Purple: ZO-1 marker, blue: nuclei, and merge (right image). All the pictures are a *z*-projection of the maximum intensity signal. **(C)** Colonization rate in laminin-coating (black) and uncoated (magenta) chips. Error bars are S.E.M.

**4. PCT WT, *Pkd1^-/-^* and *Pkd1****^+/-^* **growth rates**

We realized a doubling test to determine the proliferation rate of each cell line. To this end, all the cell types were plated at different cell densities in a 6-well plate: 5.10^3^, 10.10^3^, 20.10^3^, 40.10^3^, and 80.10^3^ cells/ml, in proliferation or differentiation medium for *Pkd1* cells. They were trypsinized and counted at different time points. For each cell type, we distinguished an exponential phase and a plateau. The duplication rate was determined during the exponential phase, between twofold cell densities: for *Pkd1^+/-^* cells, ~35h in proliferation medium and ~48 h in differentiation medium, for *Pkd1^-/-^* cells ~20 h in proliferation medium and ~25 h in differentiation medium. We noticed that the different cell lines did not proliferate at the same velocity: *Pkd1*^-/-^ cells grew faster than the control cell lines, with a two-fold increase in each medium (and the cells grew faster in proliferation medium). This was in very good agreement with the initial description of these cell lines by Wei et al^1^.

***Sup. Fig. 4.***

Doubling test on the ADPKD cellular models. Plots representing cell density over time, for the three cell lines. **(A)** PCT *Pkd1^+/-^* or *Pkd1^-/-^* cells growth in the proliferation medium. **(B)** PCT *Pkd1^+/-^* or *Pkd1^-/-^* cells growth in the differentiation medium. **(C)** PCT WT cells proliferation.

**5. Characterization of PCT *Pkd1^-/-^* and *Pkd1****^+/-^* **cells by qRT-PCR.**

The expression of transcripts for cytoskeletal proteins, adhesion proteins, and apico-basal polarity markers were compared in PCT *Pkd1^+/-^* and *Pkd1^-/-^* cells in differentiation conditions. It was also checked that the transcript level of AQP1, marker for the distal part of tubule, was very low for both cell lines (not shown), contrary to AQP2, marker for the proximal part of the tubule, thus confirming the origin of cells. Transcript levels for β-actin and the actin-binding protein vinculin were similar in both cell lines, as well as transcript levels for the tight junction protein ZO1 and the primary cilia protein Arl13b. The expression of E-cadherin and N-cadherin involved in intercellular adhesion was strongly reduced in *Pkd1^-/-^* cells compared to *Pkd1^+/-^* cells. This was in agreement with the reduced expression of E-cadherin observed in ADPKD models^2^. Some apico-basal markers were significantly less expressed in *Pkd1^-/-^* cells compared to *Pkd1^+/-^* cells: the actin-membrane linker ezrin, located to the apical pole in normal epithelial cells, and the Na^+^/K^+^ ATPase, located at the basolateral part and creating a driving force for sodium reabsorption.

***Primers:***

mGAPDH Fw AAC AGC AAC TCC CAC TCT TC

mGAPDH Rev TGG TCC AGG GTT TCT TAC TC

mACTB Fw CCTAAGGCCAACCGTGAAA

mACTB Rev GCATACAGGGACAGCACAG

mCDH1 Fw GCTTCAGTTCCGAGGTCTACA

mCDH1 Rev  GAGTCTTCCGAAAAGAAGGCTGTC

mZO1 Fw TTTCAGAGTGGGGAAACCTCC

mZO1 Rev TAGCTGCTGAACAGCAAAAGCAT

Vinculin Fw GATTACCTCATTGACGGCTCTAGG

Vinculin Rev CTCCTGCTGTCTCTCATCAATCATTT

mATP1A1 Fw CCGTGGATAACCTCTGCTTCGT

mATP1A1 Rev CGCTGTGATTGGATGGTCTCCT

mEzrin Fw GAAACTTGATAAAAAGGTCTCCGCA

mEzrin Rev TTATAATCTCCGAACTTGGCCTGAA

mAQP1 Fw CTGCTGGCGATTGACTACACTG

mAQP1 Rev GGTTTGAGAAGTTGCGGGTGAG

mAQP2 Fw CATCCTCCATGAGATTACCCCTGTA

mAQP2 Rev GGTGAAATAGATCCCAAGGAGGT

***Sup. Fig. 5:***

Quantification by qRT-PCR of gene expression for *Pkd1^+/-^* and *Pkd1^-/-^* cells. Results are expressed as the fold induction in *Pkd1^-/-^* cells (magenta) compared to *Pkd1^+/-^* cells (black) (n=2 independent experiments, each done in technical triplicates). Data points represent the 2 independent experiments and their mean. *: statistically significant difference with p<0.05.

**6. PCT WT cells do not induce tube dilation**

Tubes were seeded with PCT WT cells (n=2 chips of 5 tubes each), with or without laminin coating. The colonization over time was similar to other PCT cells (not shown). No significant tube dilation was observed with these cells.

***Sup. Fig. 6:***

**(A)** Example of time evolution of PCT WT cells in tubes, 0, 6, 16, 22 and 27 days after seeding (scale bar, 100 μm). **(B)** Kinetic evolution of mean tube diameter normalized by diameter at seeding, in function of the time after tube confluency. A blue horizontal line at D/D_0_=1, corresponding to no change in diameter, is indicated. Error bars: S.E.M. (**C)** Maximum (over time) of the mean normalized diameter. One point corresponds to one tube. Central bar, median; cross, mean; box, values between Q1 and Q3 quartiles; error bars, extreme values (between Q1-1.5*(Q3-Q1) and Q3+1.5*(Q3-Q1). (B,C) were computed only for tubes having reached full confluency during the observation period.

**7. Effects of different coatings on *Pkd1^-/-^* and *Pkd1****^+/-^* **tubes, and distribution of *Pkd1^-/-^* diameters during dilation.**

Systematic comparisons were done between tubes with or without laminin coating, with PCT *Pkd1^+/-^* and *Pkd1^-/-^* cells. Examples of chips are shown in Sup. Fig. 7. The kinetics of colonization was similar with or without laminin coating (Sup. Fig. 8A,B). Deformation data would suggest a larger tube dilation for *Pkd1^-/-^* tubes with no coating (Sup. Fig. 8C-D). Although statistically significant, this difference would have to be further confirmed by further studies, because of the small number of collagen *Pkd1^-/-^* chips (n=4). Maximal normalized tube diameters (over time) were: *Pkd1^+/-^* cells in collagen: 1.14±0.14 (mean±S.E.M.), n_tubes_=9, *Pkd1^+/-^* cells in laminin: 1.12±0.06 μm, n_tubes_=6. *Pkd1^-/-^* cells in collagen: 1.52±0.05 μm, n_tubes_=10, *Pkd1^-/-^* cells in laminin: 1.33±0.06 μm, n_tubes_=15. At last, there was still a statistically significant difference when comparing maximal deformation in 100μm spaced (laminin) chips to 200μm (all coating pooled) (p=0.01, Sup. Fig. 8E).

***Sup. Fig. 7:***

Examples of temporal evolution of tubes, without coating (collagenI) **(A)**, or with Matrigel coating **(B)**, for *Pkd1^+/-^* cells (top) and *Pkd1^-/-^* cells (bottom). Scale bar:100 μm. Days after seeding: (A) 0, 2, 7, 14, 24, (B) 0, 2, 6, 13, 24, (C,D) 0, 1, 3, 6, 11.

***Sup. Fig. 8:***

**(A-D)** 200 μm spacing chips. **(A, B)** Comparison of colonization rates of tubes without coating (collagen I, magenta) and with coating (laminin, black). A, PCT *Pkd1^+/-^* cells (collagen, n=4 chips, laminin, n=7 chips) B, PCT *Pkd1^-/-^* cells (collagen, n=4 chips, laminin, n=9 chips). Error bars: S.E.M. **(C)** Kinetic evolution of mean tube diameter normalized by diameter at seeding, in function of the time after tube confluency. A blue horizontal line at D/D_0_=1, corresponding to no change in diameter, is indicated. Each time point corresponds to 4-19 tubes for *Pkd1^-/-^* laminin, 4-13 tubes for *Pkd1^-/-^* collagen, 6-10 tubes for *Pkd1^+/-^* laminin, 2-7 tubes for *Pkd1^+/-^* collagen. Error bars: S.E.M. (**D,E)** Maximum (over time) of the mean normalized diameter. One point corresponds to one tube. Central bar, median; cross, mean; box, values between Q1 and Q3 quartiles; error bars, extreme values (between Q1-1.5*(Q3-Q1) and Q3+1.5*(Q3-Q1). **(E)** Comparison of the maximal deformation of *Pkd1^-/-^* tubes between 100μm spaced (laminin) chips and 200μm (all coating pooled). (C-E) were computed only for tubes having reached full confluency during the observation period. * indicates statistically significant difference with p<0.05.

**8. Local deformations and correlations for *Pkd1^-/-^* tubes.**

Distributions of local diameters were studied in order to evaluate diameter uniformity during *Pkd1^-/-^* tube dilation (Sup. Fig. 9A). For large diameters, the distribution of local diameters was slightly more spread, and there was a part (10-20%) of large local diameter (>150 μm), mostly corresponding to dilations at the external borders of the tubes. The kinetic evolution of the distances between tubes and some correlations are shown in Sup. Fig. 9B-D.

***Sup. Fig. 9:***

**(A)** Distributions of local diameters in order to evaluate diameter uniformity during *Pkd1^-/-^* tube dilation. All *Pkd1^-/-^* tubes, at all times were considered. For each tube, at each time, local tube diameters values were put in one of the six different tabs according to their median value: ]85 95[ μm (black), ]95 105[ μm (blue), ]105 115[ μm (cyan), ]115 125[ μm (green), ]125 135[ μm (red), ]135 145[ μm (magenta). cdf, cumulative distribution function. Note that the curves only reflect the dispersion for different median diameters, and are not intended to directly take into account the temporal evolution. **(B)** Mean distances between adjacent *Pkd1^-/-^* tubes. **(C)** Correlation coefficients between local diameters of one tube and local distances of the adjacent intertube (4 correlation coefficients corresponding to the 4 intertubes were computed for each time, and their means represented). **(D)** Correlation coefficients between adjacent contour lines of neighbour tubes. (B-D) n=8 *Pkd1^-/-^* chips, red; analysis restricted to cases where all tubes of the chips were selected for analysis (C,D) Horizontal blue line, no correlation. All coatings pooled.

**9. Cellular extensions or invasion in collagen matrix.**

Cytoplasmic extensions in the collagen around tubes (Sup. Fig. 10A), or penetration of entire cells into the collagen (Sup. Fig. 10B), were occasionally observed, both for PCT *Pkd1^+/-^* and *Pkd1^-/-^* cells, and whatever the coating (laminin or no coating). They probably reflected the degradation of collagen by cell-secreted proteases, leading to the question to know to which extent dilations were related to collagen compression and to which extend to collagen fragilization. These “invasive” phenomena were heterogeneous between chips, and different quantifications were performed in order to study if their frequency or penetration depth depended on the coating or on the cell type. **First**, attempts to quantify invasive region with our mask analysis method were done (Sup. Fig. 10C), however mean values obtained are most likely close ton analysis noise. **Second**, the maximal perpendicular penetration depth in collagen during the whole movies was manually determined. This led to the visual impression that there was no difference depending on the coating, but that *Pkd1^-/-^* cells were slightly more invasive. However, the difference between *Pkd1^-/-^* and *Pkd1^+/-^* was slightly above the limit of statistical significancy (all collagen, 42±23 μm, n=7, *vs* all laminin, 47±10 μm, n=7; all *Pkd1^+/-^*, 26±13 μm, n=14, *vs* all *Pkd1^-/-^*, 57±10 μm, n=18, p=0.076). **Third**, qualitative assessments were made by visualization of the movies (Table 1). Chips were classified with (1) no or minimal, (2) slight, (3) clear or (4) massive invasion of the surrounding collagen matrix. The percentage of chips without visible membrane protrusions or invasion was close (around 50%) for lamin coating and without coating, but was higher for *Pkd1^-/-^* cells than for *Pkd1^+/-^* cells.

This was in favor of the hypothesis that some proteases may play a role in a local fragilisation of collagen around tubes, favoring tube dilation. This phenomenon may be amplified in the case of *Pkd1^-/-^* cells, although further studies will need to confirm this point.

***Sup. Fig. 10:***

Confocal images of tubes labeled with phalloidin-TRITC (A-B, red) and with Hoechst (B, blue). **(A,B)** Cell protrusions invading the collagen matrix: **(A)** *Pkd1^-/-^* tube, images at a low resolution (10X objective). Maximal *z* projection (left) or confocal sections (right) of tubes with short cytoplasmic extensions. Bar, 100 μm. **(B)** *Pkd1^-/-^* tube, image at high resolution showing an entire cell (top) invading the collagen matrix. Bar, 50 μm. **(C)** Invasive area as determined in Sup. Fig. 2, comparisons between collagen/laminin chips with 200 μm spacing (top, blue collagen n=7, black laminin n=15; bottom, cyan *Pkd1^+/-^*, n=10, red *Pkd1^-/-^*, n=12). Because the lines in contact with Matrigel that can be the basement for invasion have dimensions on the order of 1000x5x2 μm, and because invasive areas are obtained by substraction of two manually drawn masks with possible errors of 1-2 pixels (1 pix=1.65 μm) (horizontal blue lines), the mean values obtained are in the range of error measurements, so that different approaches were considered. **(D)** Maximal perpendicular penetration depth for all chips (100 or 200 μm spacing). Left, all collagen chips (blue) *vs* all laminin chips (black). Right, all *Pkd1^+/-^* tubes (cyan) *vs* all *Pkd1^-/-^* tubes (red). A collagen *Pkd1^-/-^* chip with massive invasion was excluded from the quantitative analysis.

***Table 1:***

Visual classification of chips without or with membrane protrusions in the collagen matrix surrounding the tubes. (No or minimal, slight, clear or massive invasion). Top: number of chips. Bottom, expression in percentage.

|  | | **no or minimal** | | **slight** | | **clear** | | **massive** | | **total** |
| --- | --- | --- | --- | --- | --- | --- | --- | --- | --- | --- |
| **laminin** | | 11 | | 6 | | 4 | |  | | 21 |
| **collagen** | | 4 | | 1 | | 2 | | 1 | | 8 |
|  | |  | |  | |  | |  | | **29** |
| ***Pkd1+/-*** | | 11 | | 1 | | 2 | |  | | 14 |
| ***Pkd1-/-*** | | 7 | | 6 | | 4 | | 1 | | 18 |
|  | |  | |  | |  | |  | | **32 *** |
| (* including the chips coated with Matrigel) | | | | | | | |  | |  |
|  | | **no or minimal** | | **slight** | | **clear** | | **massive** | |  |
| **laminin** | | **52%** | | 29% | | 19% | |  | |  |
| **collagen** | | **50%** | | 12.5% | | 25% | | 12.5% | |  |
|  | |  | |  | |  | |  | |  |
| ***Pkd1+/-*** | | **79%** | | 7% | | 14% | |  | |  |
| ***Pkd1-/-*** | | **39%** | | 33% | | 22% | | 6% | |  |

**References**

1. Wei, F. *et al.* Neutrophil gelatinase-associated lipocalin suppresses cyst growth by Pkd1 null cells in vitro and in vivo. *Kidney Int* **74**, 1310–1318 (2008).

2. Charron, A. J., Nakamura, S., Bacallao, R. & Wandinger-Ness, A. Compromised cytoarchitecture and polarized trafficking in autosomal dominant polycystic kidney disease cells. *J Cell Biol* **149**, 111–124 (2000).
