# Supplementary material for: A Multitubular Kidney-on-Chip to Decipher Pathophysiological Mechanisms in Renal Cystic Diseases
Source: Front Bioeng Biotechnol. 2021 May 26;9:624553. doi: 10.3389/fbioe.2021.624553 (PMC8188354; doi:10.3389/fbioe.2021.624553)
Supplement: Supplementary file 2 [file Data_Sheet_2.PDF]

A

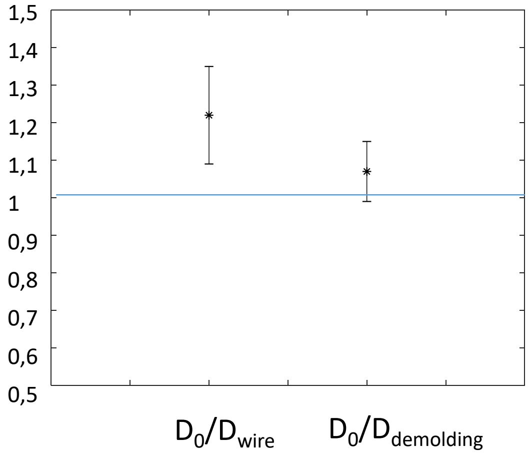

B

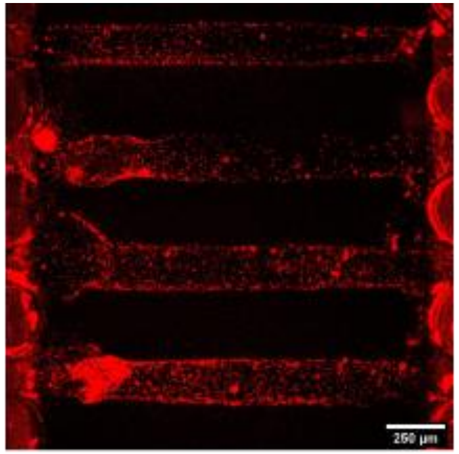

Laminin-Rhodamin

Sup. Fig. 1

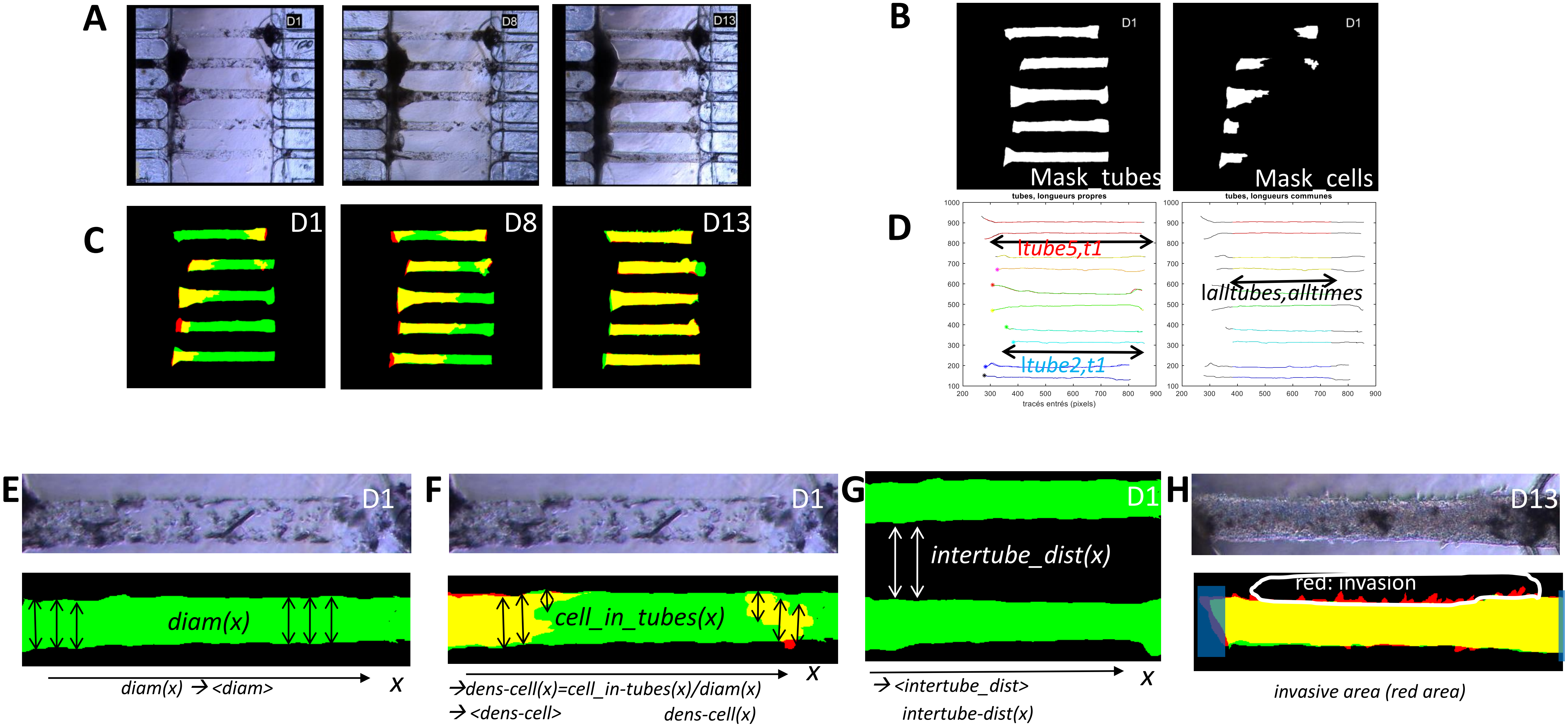
A
D1
D8
D13
B
Mask_tubes
Mask_cells
C
D
tubes, longueurs propres
tubes, longueurs communes
ltube5,t1
ltube2,t1
lalltubes,alltimes
tracés entrés (pixels)
E
diam(x)
diam(x) → <diam>
X
F
cell_in_tubes(x)
→dens-cell(x)=cell_in-tubes(x)/diam(x)
→ <dens-cell>
dens-cell(x)
G
intertube_dist(x)
→ <intertube_dist>
intertube-dist(x)
H
red: invasion
invasive area (red area)

Sup. Fig. 2

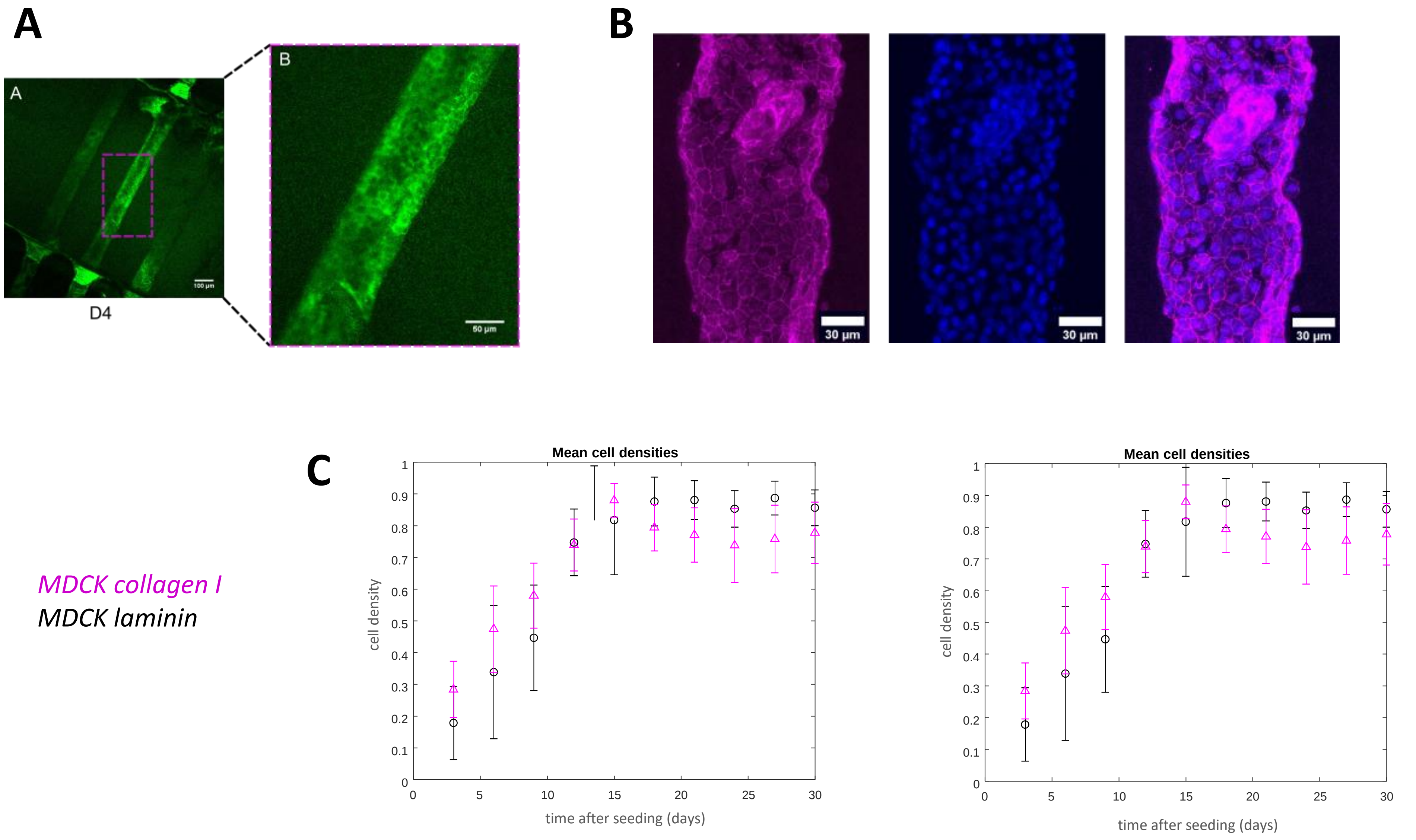
A
B
D4
50 µm
B
30 µm
30 µm
30 µm
C
MDCK collagen I
MDCK laminin
Mean cell densities
cell density
time after seeding (days)
Mean cell densities
cell density
time after seeding (days)

Sup. Fig. 3

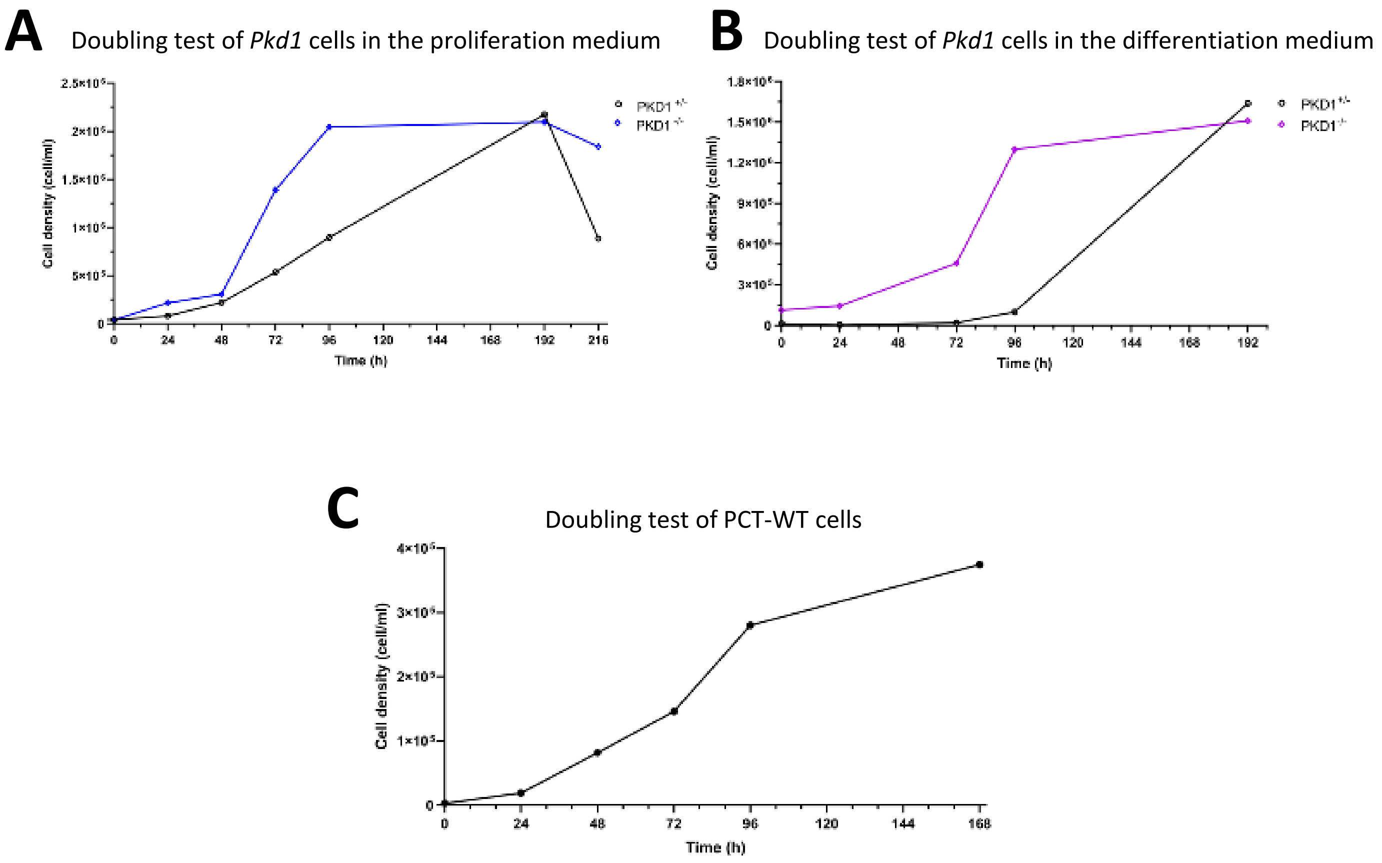
A
Doubling test of Pkd1 cells in the proliferation medium
Cell density (cell/ml)
Time (h)
0
24
48
72
96
120
144
168
192
216
PKD1 +/-
PKD1 -/-
B
Doubling test of Pkd1 cells in the differentiation medium
Cell density (cell/ml)
Time (h)
PKD1 +/-
PKD1 -/-
C
Doubling test of PCT-WT cells
Cell density (cell/ml)
Time (h)

Sup. Fig. 4

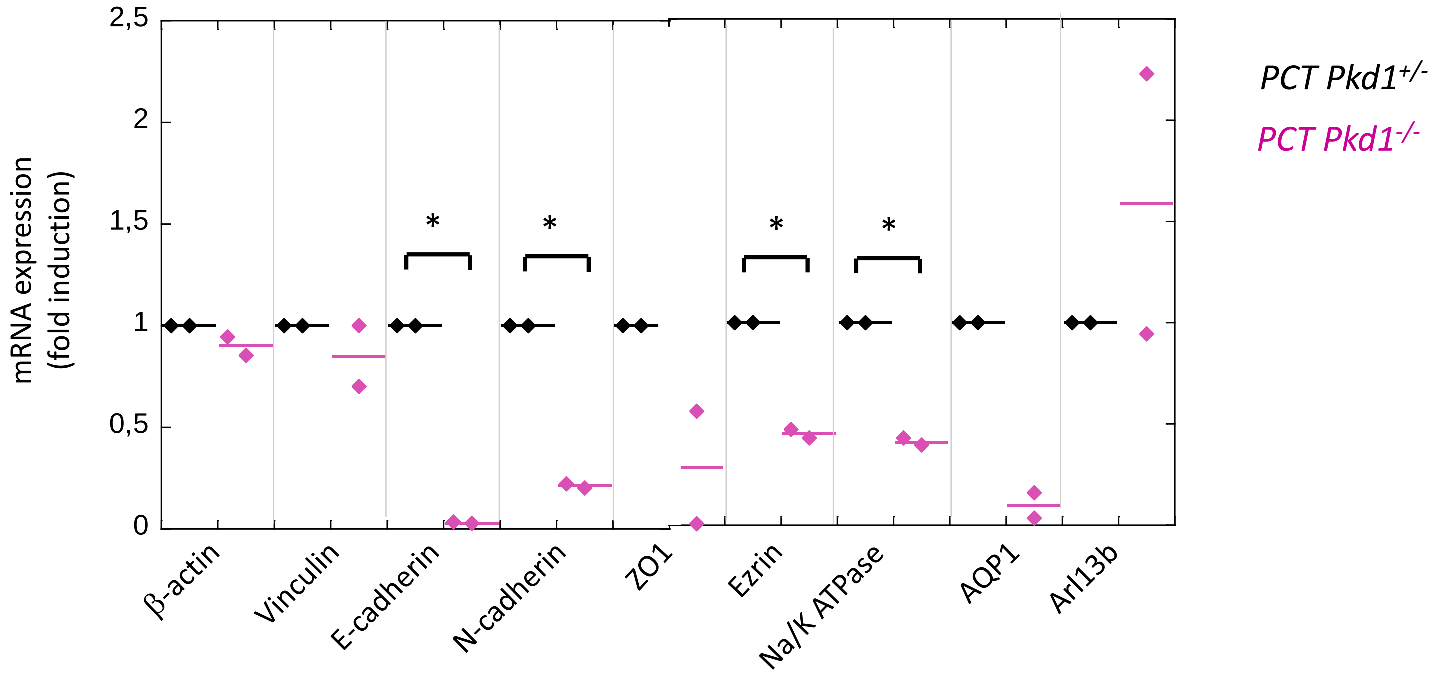
2,5
2
1,5
1
0,5
0
mRNA expression
(fold induction)
*
*
*
*
β-actin
Vinculin
E-cadherin
N-cadherin
ZO1
Ezrin
Na/K ATPase
AQP1
Arl13b
PCT Pkd1+/-
PCT Pkd1-/-

Sup. Fig. 5

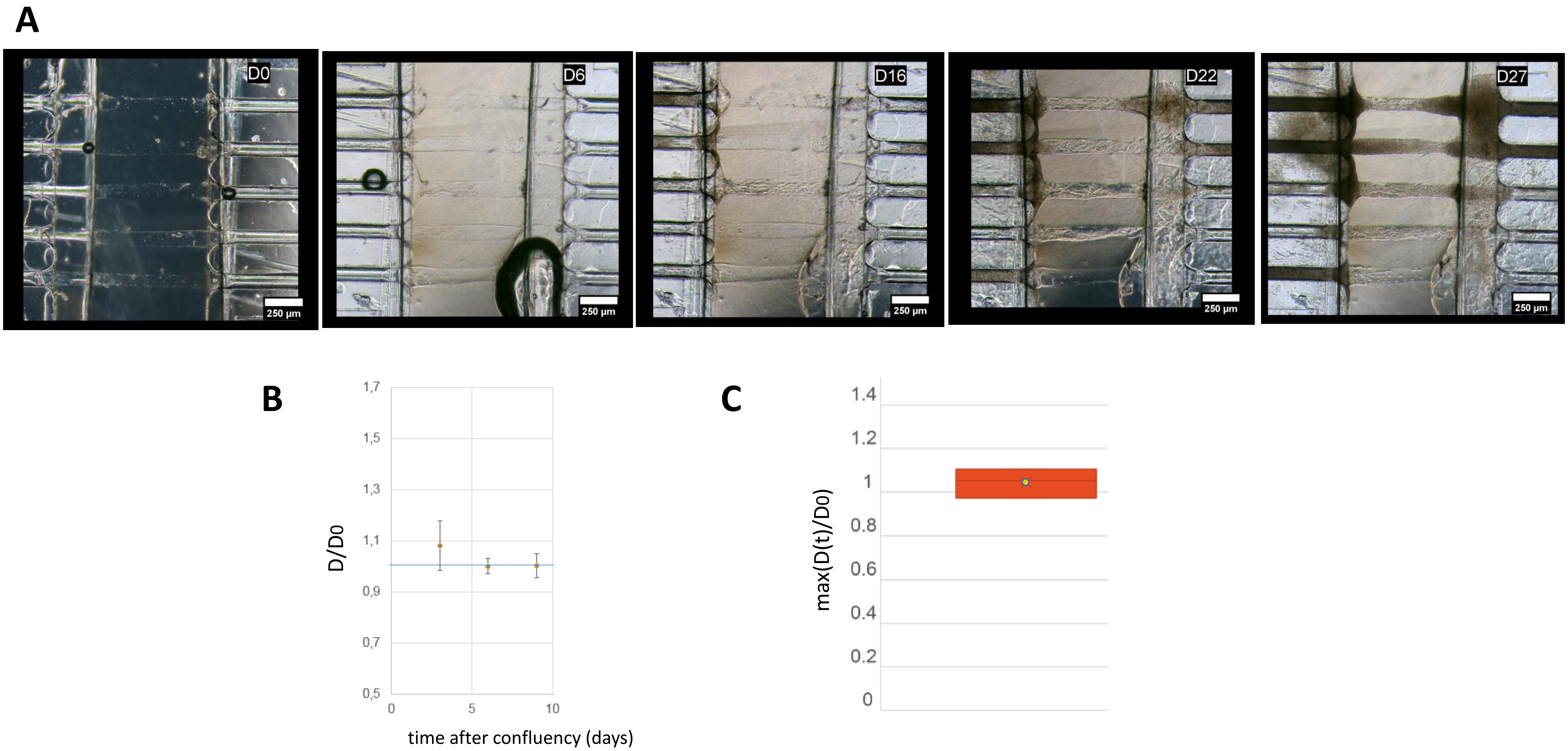
A
D0
D6
D16
D22
D27
250 µm
B
D/D0
1,7
1,5
1,3
1,1
0,9
0,7
0,5
0
5
10
time after confluency (days)
C
max(D(t)/D0)
1.4
1.2
1
0.8
0.6
0.4
0.2
0

Sup. Fig. 6

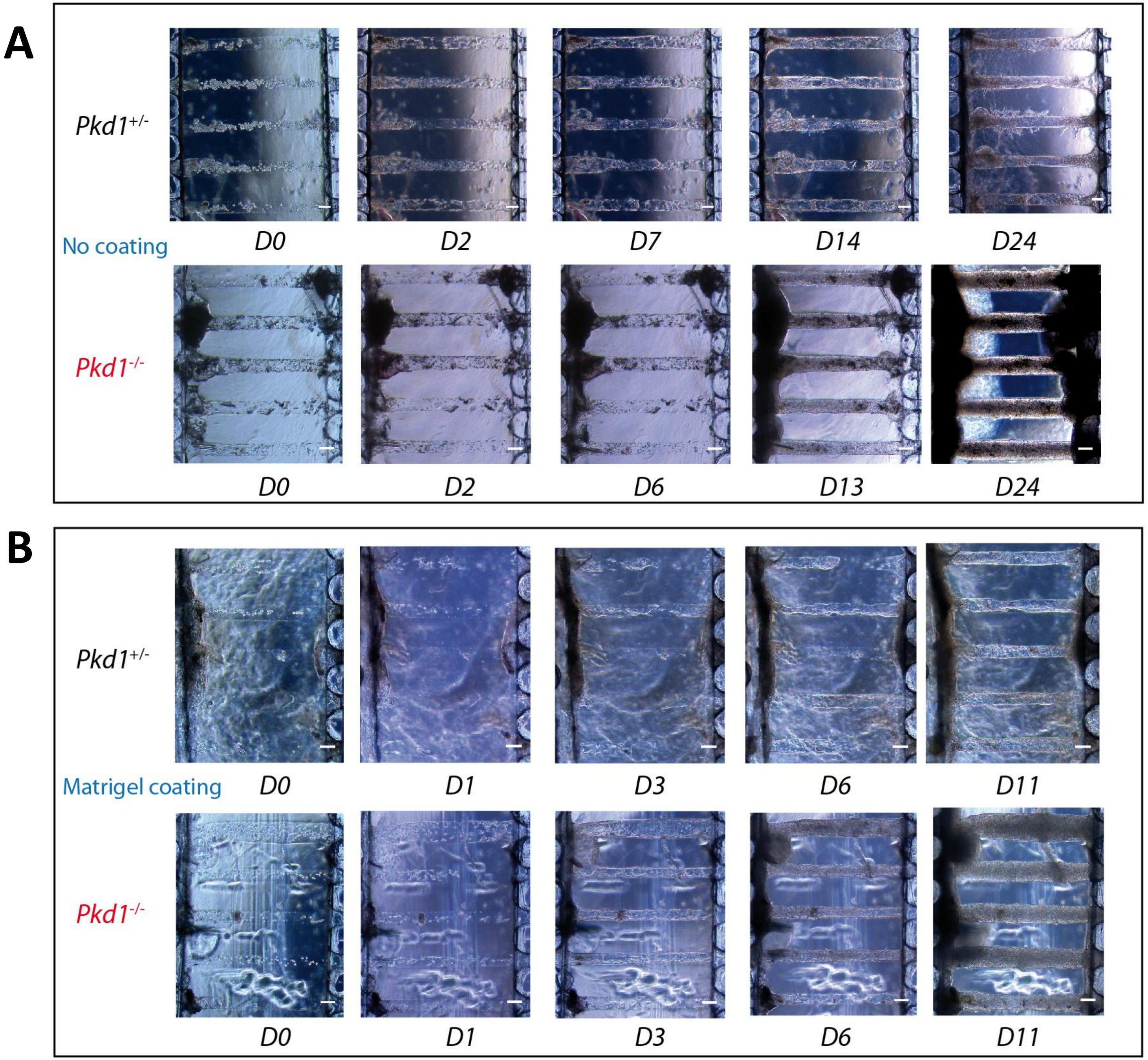
A
Pkd1+/-
No coating
D0
D2
D7
D14
D24
Pkd1-/-
D0
D2
D6
D13
D24
B
Pkd1+/-
Matrigel coating
D0
D1
D3
D6
D11
Pkd1-/-
D0
D1
D3
D6
D11

Sup. Fig. 7

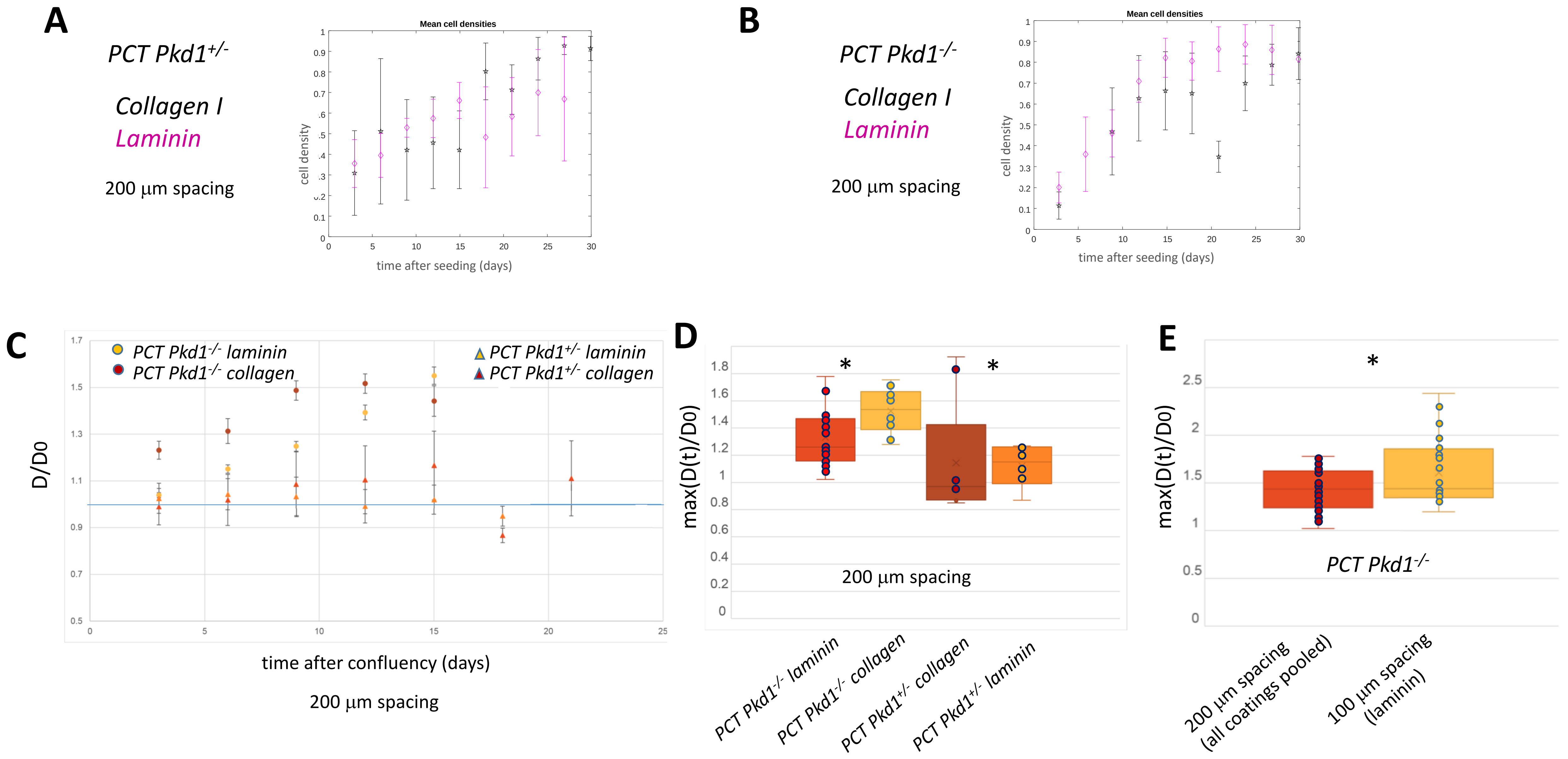
A
PCT Pkd1+/-
Collagen I
Laminin
200 µm spacing
Mean cell densities
cell density
time after seeding (days)
B
PCT Pkd1-/-
Collagen I
Laminin
200 µm spacing
Mean cell densities
cell density
time after seeding (days)
C
PCT Pkd1-/- laminin
PCT Pkd1-/- collagen
PCT Pkd1+/- laminin
PCT Pkd1+/- collagen
D/D0
time after confluency (days)
200 µm spacing
D
max(D(t)/D0)
200 µm spacing
PCT Pkd1-/- laminin
PCT Pkd1-/- collagen
PCT Pkd1+/- collagen
PCT Pkd1+/- laminin
E
max(D(t)/D0)
PCT Pkd1-/-
200 µm spacing (all coatings pooled)
100 µm spacing (laminin)

Sup. Fig.8

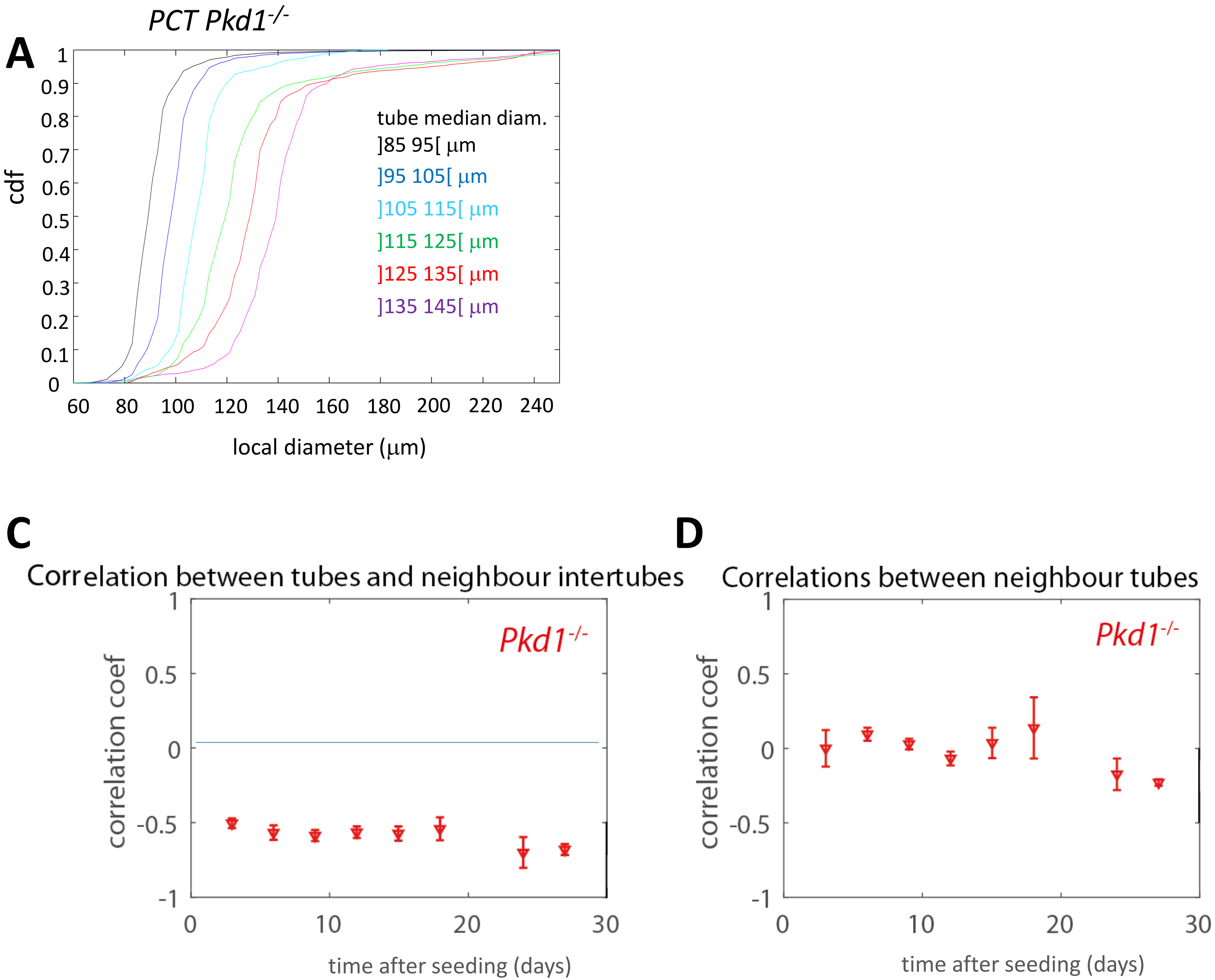
PCT Pkd1-/-
A
cdf
local diameter (µm)
tube median diam.
]85 95[µm
]95 105[µm
]105 115[µm
]115 125[µm
]125 135[µm
]135 145[µm
B
Mean distance between tubes
Pkd1-/-
D (um)
time after seeding (days)
C
Correlation between tubes and neighbour intertubes
Pkd1-/-
correlation coef
time after seeding (days)
D
Correlations between neighbour tubes
Pkd1-/-
correlation coef
time after seeding (days)

Sup. Fig.9

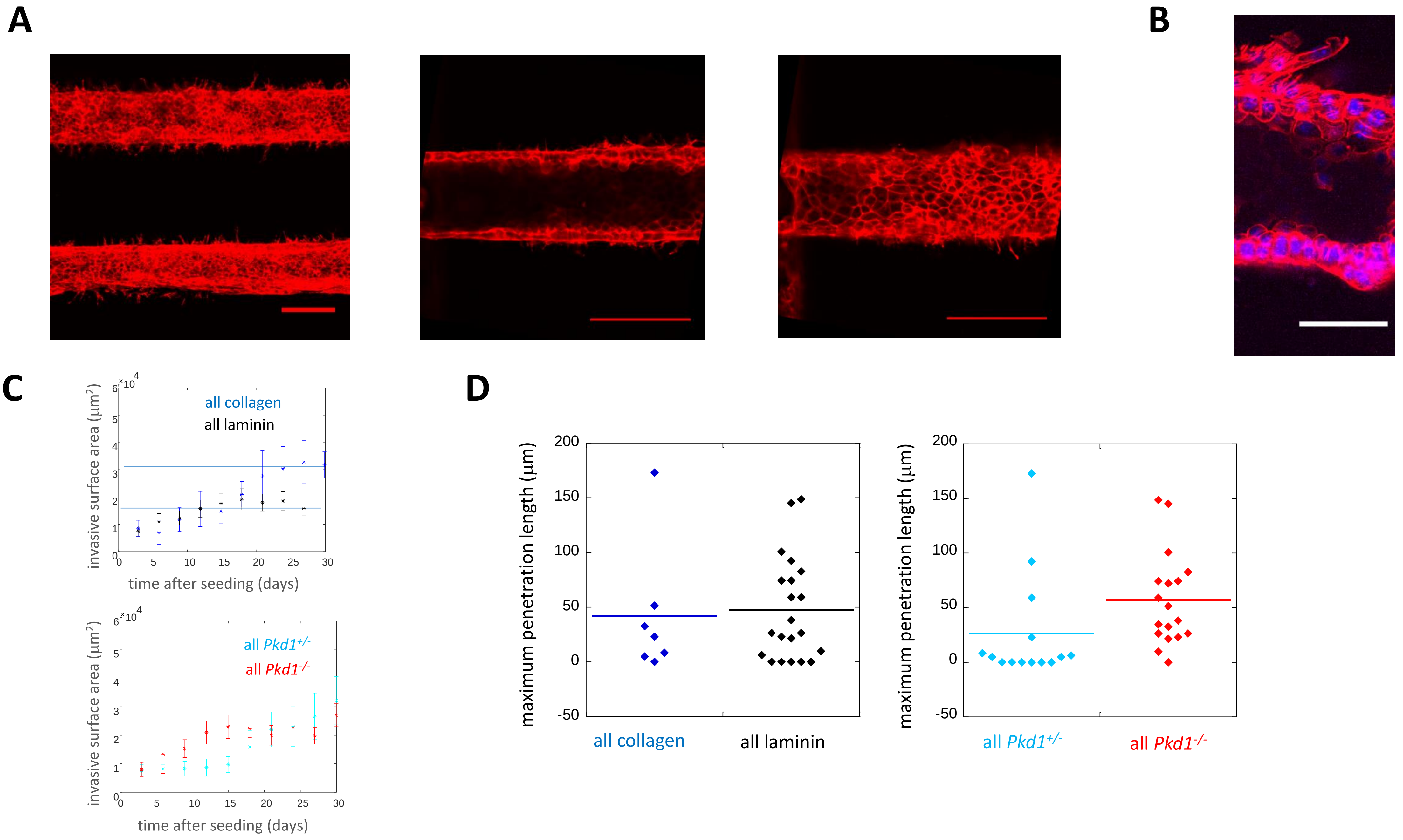
A
B
C
6
×10^4
5
4
3
2
1
0
0
5
10
15
20
25
30
invasive surface area (μm²)
time after seeding (days)
all collagen
all laminin
all Pkd1+/-
all Pkd1-/-
D
200
150
100
50
0
-50
maximum penetration length (μm)
all collagen
all laminin
all Pkd1+/-
all Pkd1-/-

Sup. Fig. 10
